# Adult life-course trajectories of psychological distress and economic outcomes in midlife during the COVID-19 pandemic: evidence from the 1958 and 1970 British birth cohorts

**DOI:** 10.1007/s00127-022-02377-w

**Published:** 2023-01-19

**Authors:** V. Moulton, A. Sullivan, A. Goodman, S. Parsons, G. B. Ploubidis

**Affiliations:** Centre for Longitudinal Studies, University College London, London, UK

**Keywords:** COVID-19, Psychological distress, Life-course trajectories, Economic shock, Economic inequalities, Debt, Unemployment

## Abstract

**Purpose:**

Financial adversity in times of economic recession have been shown to have an unequal effect on individuals with prior mental health problems. This study investigated the relationship between mental health groupings across the adult life-course and change in financial situation and employment status during the COVID-19 pandemic, as well as the use of financial measures to mitigate the economic shock.

**Methods:**

Using two nationally representative British birth cohorts, the National Child Development Study (1958) *n* = 17,415 and 1970 British Cohort Study *n* = 17,198, we identified 5 different life-course trajectories of psychological distress from adolescence to midlife which were similar but not identical across the two cohorts. We explored their relation to changes in financial and employment circumstances at different stages during the pandemic from May 2020 to March 2021, applying multinomial logistic regression and controlling for numerous early life covariates, including family socio-economic status (SES). In addition, we ran modified Poisson models with robust standard errors to identify whether different mental health trajectories were supported by government and used other methods to mitigate their financial situation.

**Results:**

We found that the financial circumstances of pre-pandemic trajectories of psychological distress with differential onset, severity, and chronicity across the life-course were exacerbated by the COVID-19 economic shock. The ‘stable-high’ (persistent severe symptoms) and ‘adult-onset’ (symptoms developing in 30s, but later decreasing) groups were vulnerable to job loss. Compared to pre-pandemic trajectory groupings with no, minor, or psychological distress symptoms in early adulthood, the ‘stable-high’, ‘midlife-onset’ (symptoms developing in midlife), and ‘adult-onset’ trajectory groups were more likely to seek support from the UK governments economic response package. However, trajectories with pre-pandemic psychological distress were also at greater risk of reducing consumption, dis-saving, relying on increased financial help from family and friends, and also taking payment holidays (agreements with lenders to pause mortgage, credit card or loan payments for a set period) and borrowing.

**Conclusion:**

This work highlights different trajectories of pre-pandemic psychological distress, compared to groups with no symptoms were more vulnerable to pandemic-related economic shock and job loss. By adopting unsustainable mitigating measures (borrowing and payment holidays) to support their financial circumstances during COVID-19, these mental health trajectories are at even more risk of lasting adverse impacts and future economic difficulties.

**Supplementary Information:**

The online version contains supplementary material available at 10.1007/s00127-022-02377-w.

## Introduction

At the start of the COVID-19 pandemic, there were warnings that those with prior mental health problems may face a greater risk of unemployment and financial hardship [1, 2]. Previous research on pandemics and emergencies (e.g., natural disasters) has shown that such events often have a greater impact on socially disadvantaged groups [3]. Additionally, financial adversity in times of economic recession has been shown to have disparate effects on individuals according to their prior mental health status [4, 5], including an unemployment penalty associated with pre-existing psychological distress [6].

The COVID-19 economic downturn in the UK in 2020 and early 2021 differs from most recent recessions in that the effect on the labor market was immediate, the reduction in economic activity resulted in reduced working hours or temporary/permanent termination of employment in ‘non-essential’ occupations. In response, to insure households against economic shock, the UK government introduced a number of economic measures. These included the Job Retention Scheme (JRS), whereby 80% of a furloughed employee’s wages (up to £2500 per month) was paid by the government. In addition, some social security payments, a £20 weekly increase to Universal Credit (UC) and the Working Tax Credit (WTC) were introduced, as well as the ability to apply for temporary payment deferrals on mortgages, rent, council tax, credit cards and personal loans.

Despite the UK governments response package, studies have found individuals were differentially exposed to the economic impact of the COVID shock in the UK, owing to individual characteristics, lower incomes, types of work, and different private and public support mechanisms utilized [7–9]. Emerging research from the COVID-19 pandemic suggests that these factors could possibly have a greater impact on individuals with poorer pre-pandemic mental health. An investigation of 12 longitudinal studies in the UK general population, found that poor pre-pandemic mental health (captured at one time-point) was associated with 5–13% greater odds of economic disruption, including loss of employment and income during COVID-19 [10].

Indeed, people with poor mental health compared to the general population tend to experience a higher prevalence of poverty, unemployment, underemployment, and dependency on public benefits [11–13]. The direction of causation has not been clearly defined, whether the relationship is better explained by social causation, social selection or both [14, 15]. The social causation hypothesis proposes that adverse social and economic disadvantage (such as financial stress, increased adverse life events such as negative income shocks, lower education, food insecurity, income insecurity, and reduced economic resources) increase risk for mental illness. Conversely, the social selection/drift hypothesis proposes that predisposition to and people living with mental illness either prevents the attainment of higher socio-economic status (SES) by impeding educational attainment and maintaining gainful employment, resulting in reduced income; or poor mental health precipitates a downward shift into poverty, through for example reduced economic productivity, increased stigma, and reduced access to society’s opportunity structures and institutions. A difficulty in determining the direction of causation is that both SES and mental health are influenced by family background, and both evolve dynamically, sometimes in parallel or retroactively over the life-course and are, therefore, not mutually exclusive. Existing evidence supports both social selection and social causation explanations [14–16].

Socioeconomic inequalities are associated with mental health problems in childhood and adolescence, children from socioeconomically disadvantaged families are more likely to develop mental health problems than their peers from socioeconomically advantaged families [17]. Few studies have examined the influence of early life-course mental health on economic outcomes, perhaps because of the bidirectional and mutually reinforcing nature of the relationship, as well as the availability of suitable data [15, 18]. However a small body of work has focused on the long-term impact of child and adolescent mental health, on family income [19, 20], unemployment [21], and earnings in adulthood [19, 22]. In particular, Goodman et al. (2011) using data from the 1958 National Child Development Study (NCDS) found that psychological problems experienced by the age of 16 were associated with a 28% lower household income by age 50, while controlling for childhood socio-economic status (SES). Also, Smith and Smith (2010) compared siblings, thus controlling for unobserved family and neighborhood effects using the American Panel Study of Income Dynamics found the impacts of childhood psychological problems on adult socio-economic status were large.

Also, poor mental health during adulthood may shape future economic consequences, such as worsened labor market outcomes later in life. After a diagnosis of depression or anxiety, employment rates and incomes have been estimated to fall by as much as half, relative to those with no symptoms [23]. Vulnerabilities are also exacerbated by rapidly worsening economic conditions, such as those experienced during COVID-19. For example, a study examining the impact of the ‘Great Recession’ on employment for persons with mental illnesses in 27 European countries found unemployment was greater before and after the recession and increased more steeply, for individuals with mental illness [5]. In addition, post the ‘Great Recession’ in the United States, mental health problems were associated with poorer employment outcomes as well as other negative economic outcomes including lower wage income and a greater dependency on food stamps [13].

Regarding studies conducted during the COVID-19 pandemic, prior mental health has mostly been measured at one time-point only, thereby possibly underestimating the influence of poor mental health at different stages of the life-course [24, 25]. Furthermore, psychological distress in the general population has been shown to be heterogenous, following different longitudinal trajectories that vary in terms of age of onset, symptom severity and risks of recurrence [26–28]. Specifically, the age of onset of generalized anxiety disorder (median age 24–50) and mood disorders (median age 29–40) [29] are in adulthood, often during the early stages of an adult’s work career and into the prime of their economic lives, and could, therefore, possibly hinder human capital accumulation [30]. In addition, stigma and discrimination associated with mental health [31] and an individual’s beliefs about their own abilities and decision making [32, 33] may further exacerbate adverse economic outcomes. Moreover, financial hardship and psychological distress seem to have a reciprocal relationship that creates a cycle of socio-economic decline and mental health deterioration [15, 16, 18].

To the best of our knowledge, no study has investigated the relationship between differential mental health across the adult life-course and economic outcomes after the COVID-19 economic shock. In this study, we examine trajectories of psychological distress from adolescence to midlife, thus giving an overview of the timing of onset along with the severity and chronicity at different life-stages. We investigate how these trajectories were associated with economic outcomes and changes in financial situations following the sudden reduction in economic activity at the start of the pandemic in 2020, thus distinguishing the relationship between the timing of onset and severity of pre-pandemic life-course psychological distress and adverse economic outcomes in later life as a result of the COVID-19 economic shock. We employ two large nationally representative British birth cohorts, the NCDS (1958) and BCS70, both in midlife when the pandemic started to examine whether,

1. after the economic shock distinct psychological distress trajectories were more at risk of changes in their financial and employment situation at three time-points during the pandemic,
2. and if they were more likely to have been supported by the governments COVID-19 economic mechanisms, as well as taking other measures to mitigate the change in their economic situation during the pandemic (March 2020–March 2021).

At the start of the pandemic, the NCDS were in their early 60s, some cohort members had already or were transitioning from work to retirement, while the BCS70 were in their early 50s—most still working and potentially more exposed to changes in the labor market. Also, although there are considerable disparities in wealth in the UK, wealth tends to follow a life-cycle pattern, peaking for households in later middle adulthood [34], suggesting this age-group, in particular the NCDS could have accrued financial resources ensuring greater resilience to economic shocks, than younger cohorts. As well as age effects, there are cohort and period effects. Both cohorts have lived through a number of recessions, 1980–83, 1990–93 and the ‘Great Recession’ in 2008, and seen vast technological transformation, and restructuring of employment from traditional manufacturing to the service sector [35].

This study, by taking a life-course approach investigates pre-pandemic mental health at more than one time-point, looks at mental health across adulthood and treats mental health as heterogenous, thereby looking at the relation between mental health in terms of varying age of onset, symptom severity and recurrence across adulthood and the association with financial circumstances during the pandemic. In addition, we use data from two cohorts both in middle-life during the COVID-19 pandemic, but possibly at different developmental stages in their economic and employment life-cycles.

## Methods

### Participants

Our data are from two ongoing cohort studies:

*1958 National Child Development Study*: The NCDS follows the lives of 17,415 people that were born in England, Scotland or Wales in a single week in March 1958. The NCDS started in 1958 as the Perinatal Mortality Survey and captured 98% of the total births in Great Britain in the target week. The cohort has been followed up ten times between ages 7 and 55 [36, 37].

*1970 British Cohort Study*: The BCS70 follows the lives of 17,198 people (representing 95% to 98% of the target population) born in England, Scotland and Wales in a single week in April 1970. Participants have since been followed up nine times between ages 5 and 46 [38, 39].

In addition, during the COVID-19 pandemic participants of the NCDS and BCS70 completed a web survey at three different time-points when they were aged 62 and 50, respectively. The first survey was conducted during the first national lockdown, between 4 and 26 May 2020 (Wave 1: NCDS N: 5,178; BCS N: 4,223), the second between 10 September and 16 October (Wave 2: NCDS N: 6,282; BCS N: 5,320; when the first national lockdown had been lifted, but restrictions on social contact still remained, and the third survey during the third national lockdown, between 1 February and 21 March 2021 (Wave 3: NCDS N: 6,757; BCS70 N:5,684) [40].

Our analytic sample included all participants in the NCDS and BCS70 surveys, excluding those who had died or emigrated by age 50 in the NCDS (*n* = 15,291) and age 46 in the BCS70 *n* = 16,128 (sample descriptives in Table S1 in the supplement). To deal with attrition and item non-response and to restore sample representativeness, we used multiple imputation (MI) with chained equations, imputing 25 data sets [41] separately for both cohorts at each wave of the COVID-19 surveys. All variables used in our main analysis, as well as a set of auxiliary variables were included in the imputation models to maximize the plausibility of the ‘missing at random’ (MAR) assumption to reduce bias due to missing data [37, 42]. As an additional sensitivity analysis all models were rerun in line with the ‘impute and delete’ method [43] and the main findings did not differ (see Tables S2a and S2b).

### Measures

#### Trajectories of pre-pandemic psychological distress

Psychological distress was measured in both cohorts with the nine-item version of the Malaise Inventory [44, 45] from ages 23 (Chronbach’s Alpha (α) = 0.69), 33 ((α) = 0.73), 42 ((α) = 0.74) and 50 ((α) = 0.79) in the NCDS and ages 26 ((α) = 0.71), 34 ((α) = 0.76), 42 ((α) = 0.77) and 46 ((α) = 0.81) in the BCS70. The Inventory ranges from 0 to 9, a higher score identifying greater psychological distress. Psychological distress captures depression and anxiety symptoms [46]. In both surveys, the Malaise items were assessed via written self-completion, either on paper or via computer. The Malaise Inventory has been shown to have good psychometric properties [47], measurement invariance [48], and has been used in general population studies as well as investigations of high risk groups [49]. In both cohorts, at age 16 four items (ranging from 0 to 4, a higher score relates to greater psychological distress, ((α) = 0.50 in the NCDS and (α) = 0.60) in the BCS70) from the Childrens' Behavior Questionnaire reflective of affective disorders (Low mood, irritability, worry, and fearfulness), as reported by the child’s mother were employed [45]. We used latent variable mixture models to identify five longitudinal typologies of psychological distress in both cohorts as the most parsimonious models [26].

**Fig. 1:**
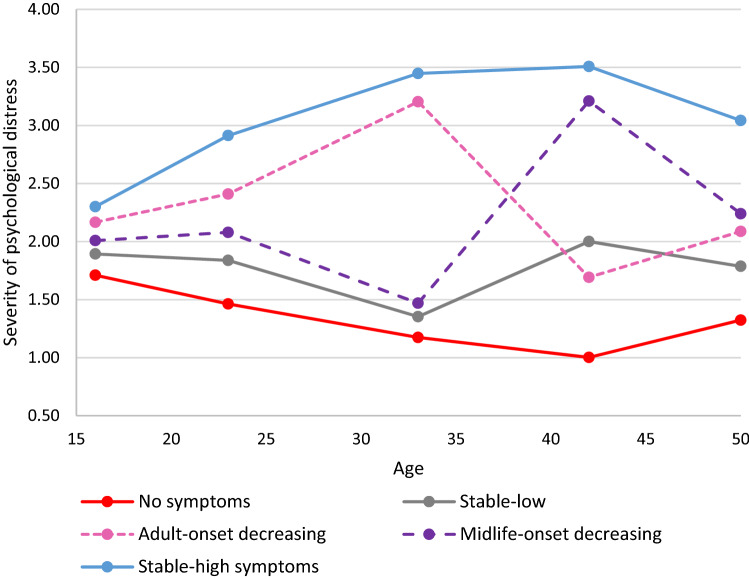
Five longitudinal classes of psychological distress from age 16 to 50 in the NCDS (*n* = 11,579)

**Fig. 2:**
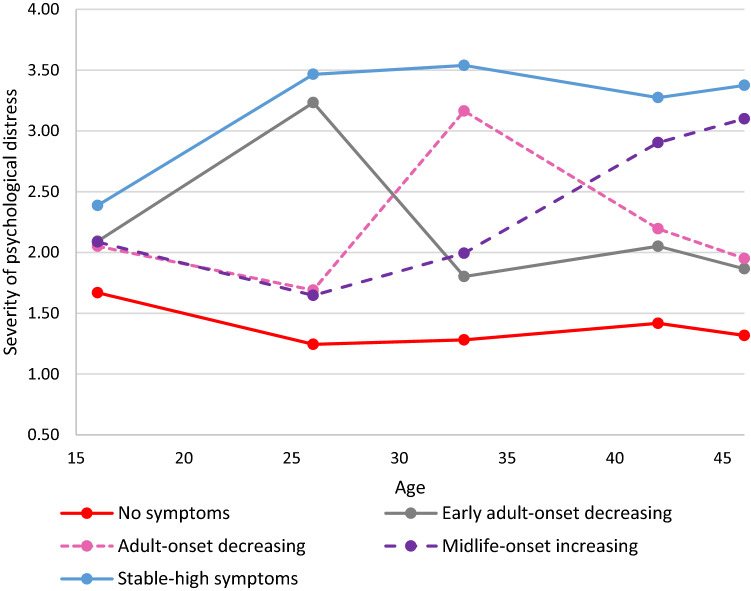
Five longitudinal classes of psychological distress from age 16 to 46 in the BCS70 (*n* = 10,236)

Figures 1, 2 show the means for each longitudinal latent class on each of the five measures (for results see Table S3a–c). Although, the five trajectories were not identical in the two cohorts, some of the groupings were very similar. In both cohorts, the largest trajectory had few or no symptoms, ‘no symptoms’. Both cohorts also had a trajectory with persistent severe symptoms ‘stable-high symptoms’, and a trajectory with few symptoms in adolescence/early adulthood with adult onset (early 30s) and favorable outcomes ‘adult-onset decreasing’. Both cohorts also had a trajectory with symptoms developing in midlife; in the NCDS, the outcome was more positive ‘midlife-onset decreasing’ and in the BCS70, the symptoms remained severe ‘midlife-onset increasing’. The final trajectory in the NCDS repeated minor symptoms ’stable-low’ symptoms, while in the BCS70, the final trajectory had symptoms in early adulthood, but not in adolescence or midlife ‘early-adult onset decreasing’.

#### Outcomes

There were two outcomes capturing change in economic circumstances asked at three time-points during the COVID-19 pandemic: change in financial situation and change in employment circumstance from pre- to during COVID-19. *Change in financial situation* was assessed, ‘overall how do you feel your current financial situation compares to before the coronavirus outbreak’; much worse off, a little worse off, about the same (reference category) or a little/ much better off in May 2020, Sept/Oct 2020, and Feb/March 2021. *Change in employment status* was derived by asking for employment status just before the first coronavirus outbreak, and during the pandemic. At each time-point, a variable was constructed to identify stability and change in employment throughout the pandemic (March–May 2020, March–Sept/Oct 2020, and March 2020–Feb/March 2021) as follows: in work pre-pandemic and stayed in work ‘work–work’, in work pre-pandemic and was furloughed ‘work–furlough’, in work pre-pandemic and was made unemployed ‘work–not work’, and other groups (e.g., no change ‘retired–retired’, ‘unemployed–unemployed’ and change, e.g., ‘work–retired’).

In addition, we captured a variety of methods used to mitigate the economic shock: at any time (from March 2020 to March 2021) made any new benefit claims, taken payments holidays since the outbreak, borrowed, reduced consumption or used savings*. New benefit claims* were assessed at each wave by asking, ‘since the coronavirus outbreak have they or their partner made any new claims for any of the following: free school meals, Universal Credit (UC), Employment and Support Allowance (ESA), Statutory Sick Pay (SSP), council tax support or reduction, carers allowance or personal independence payments, new governmental financial support for self-employed people, or not. These were combined to create a single variable, any new claims from 27 March 2020 to 21 March 2021, or not. *Payment holidays* was examined by asking at each wave ‘since the coronavirus outbreak had they used any mortgage, rent, council tax, or interest payment holidays or other debt repayments’. These were amalgamated into a single variable, taken any payment holidays from the end of March 2020 to March 2021, or not. Also*, increase in financial help* was assessed by asking at waves 2 and 3 ‘since the coronavirus outbreak in March 2020, have (they or their partner) received financial help, in the form of money or by paying for goods (for example groceries, medicines) from…family and friends’, and if so was this an increase since the outbreak in March 2020. These questions were combined into a single variable, financial help increased from the end of March 2020 to March 2021, or not. Other mitigation strategies were examined by asking ‘You said that you are worse off now compared to before the coronavirus outbreak in March 2020. Have you (or your partner) done any of the following as a result of this…’. These were transformed into dichotomous variables; reduced spending or not; used savings or not; new borrowing from bank or credit card or not; and new borrowing from family and friends or not, from the end of March 2020 to March 2021. Also, to examine the potential impact of the removal of the £20 UC uplift, we created a variable ‘claiming UC or not’ based on all making a UC claim from January 2020 to March 2021.

#### Potential confounders

We include in our analysis a rich set of variables comprising early life factors (sex, ever breastfed, mother smoked daily during pregnancy, gestation period, and birthweight), socio-economic factors (at one time-point: parental social class, education, housing tenure, access to amenities, total household income, marital status, and crowding at three time-points), parental factors (maternal age at birth, mother worked at all in first five years, and separated from child), child behavior and health (cohort member bedwetting since age 5, had any medical conditions at 7/5, Body Mass Index (BMI) at age 11/10), and cognitive ability at age 7/5 and 11/10 (details are available in supplementary Table S4).

### Analytic approach

To answer question 1, we used the psychological distress profiles to explore their relation to changes in financial and employment circumstances at three time-points during the coronavirus outbreak, applying multinomial logistic regression and controlling for numerous pre-adult covariates. In addition, we ran modified Poisson models with robust standard errors that return risk ratios for ease of interpretation and to avoid bias due to non-collapsibility of the odds ratio [50] to identify whether differential life-course trajectories of psychological distress were more likely to have been supported by the benefit system, payment holidays, borrowing and other methods of mitigating the economic shock during the pandemic, thereby answering question 2.

## Results

### Pre-pandemic psychological distress trajectories

In our analytic sample (Table S1), in the NCDS 40.9% (95% CI  40.2, 42.1) had ‘no symptoms’ of psychological distress across the life-course, while 19.9% (95% CI 19.1, 20.6) had ‘stable-high’ symptoms, 16.4% (95% CI 15.7, 17.0) ‘stable-low’, 11.9% (95% CI 11.1, 12.6) ‘midlife-onset’ decreasing, and 10.8% (95% CI 10.1, 11.6) ‘adult-onset’ decreasing. In the BCS70, half (52.8%, (95% CI 51.5, 54.1) had ‘no symptoms’, 19.3% (95% CI 18.1, 20.5) ‘stable-high symptoms’, 11% (95% CI 9.9, 12.2) ‘adult-onset’ decreasing, 9.1% (95% CI 8.4, 9.8) ‘early-adult onset’ decreasing, and 7.8% (95% CI 7.2, 8.4) ‘midlife-onset’ increasing.

### Descriptive statistics of psychological distress trajectories and financial circumstances during the COVID-19 pandemic

As shown in Table 1 during the pandemic, around half (NCDS: 51.4–59.5% (95% CI 47.5–56.9, 55.2–62.1)); BCS70: 42.0–53.4% (95% CI 38.9–51.0, 45.2–55.8)) of participants’ financial situations were similar to pre-pandemic circumstances. However, any change in circumstances from pre to during the pandemic were proportionally greater for those with worsening finances; while for a smaller number, their financial circumstances improved during the crisis. A year into the pandemic, a higher proportion of the ‘stable-high’ (19%, 95% CI 15.8–22.3) and the ‘midlife-onset’ (15.5%, 95% CI 12.6–18.4) trajectories in the NCDS were much worse off financially than pre-COVID, compared to the ‘no symptoms’ (10.7%, 95% CI 9.3–12.2) trajectory. In the BCS70, the ‘stable-high’ group (W1 20.4%, 95% CI 16.0, 24.9; W2 18.8%, 95% CI 13.0, 24.7; W3 18.1%, 95% CI 14.1, 22.1) were much worse off throughout the pandemic and a year on the ‘midlife-onset’ group (W3 15.6%, 95% CI 11.9, 19.2) were also much worse off, compared to the ’no-symptoms’ (W1 12.7%, 95% CI 10.7, 14.7; W2 7.7%, 95% CI 6.0, 9.4; W3 9.6%, 95% CI 7.7, 11.5) group.

**Table 1:** Distribution of cohort members financial circumstances during the COVID-19 crisis by pre-pandemic psychological distress trajectories

	NCDS (*n* = 15,291)					Total %	BCS70 (*n* = 16,128)					Total %
Outcomes pre- and during COVID	No symptoms %	Stable low %	Adult onset %	Midlife onset %	Stable high %		No symptoms %	Early-adult onset %	Adult onset %	Midlife onset %	Stable high %	
Current financial situation (May 2020)												
Better off	11.9 [10.4, 13.5]	11.7 [9.3, 14.0]	10.6 [8.0, 13.1]	10.6 [8.1, 13.0]	7.4 [5.1, 9.8]	10.7 [9.4, 12.0]	19.1 [17.0, 21.3]	16.9 [13.6, 20.2]	19.1 [15.2, 23.0]	18.0 [14.7, 21.3]	13.3 [9.6, 17.0]	17.7 [15.7, 19.7]
About the same	51.8 [48.5, 55.1]	51.5 [47.3, 55.7]	49.7 [43.9, 55.5]	51.3 [46.0, 56.6]	51.2 [43.7, 58.7]	51.4 [47.5, 55.2]	43.8 [40.7, 46.9]	46.4 [40.5, 52.3]	38.3 [32.4, 44.2]	40.4 [34.8, 46.2]	37.9 [31.4, 44.4]	42.0 [38.9, 45.2]
A little worse off	25.2 [22.4, 28.1]	25.1 [21.9, 28.3]	23.6 [17.9, 29.3]	26.3 [21.1, 32.4]	24.5 [19.7, 29.4]	25.0 [22.1, 28.0]	24.4 [21.7, 27.0]	20.8 [16.1, 25.4]	28.4 [21.9, 34.9]	28.0 [23.0, 33.1]	28.3 [23.0, 33.5]	25.5 [23.0, 28.1]
Much worse off	11.0 [8.4, 13.6]	11.7 [8.3, 15.1]	16.1 [11.8, 20.4]	11.9 [8.2, 15.6]	16.8 [10.9, 22.8]	12.9 [10.3, 15.6]	12.7 [10.7, 14.7]	15.9 [12.1, 19.7]	14.2 [9.4, 19.1]	13.5 [9.1, 17.9]	20.4 [16.0, 24.9]	14.7 [12.7, 16.7]
Current financial situation (Sep/Oct 2020)												
Better off	13.8 [12.2, 15.4]	11.8 [10.1, 13.5]	10.4 [8.1, 12.7]	10.2 [8.2, 12.3]	8.3 [6.6, 10.1]	11.6 [10.5, 12.7]	19.7 [17.9, 21.5]	14.2 [11.6, 16.8]	16.9 [13.3, 20.4]	16.2 [13.2, 19.2]	10.6 [8.0, 13.1]	16.9 [15.3, 18.4]
About the same	60.6 [57.8, 63.4]	60.3 [56.6, 64.1]	59.4 [54.7, 64.1]	60.4 [56.4, 64.2]	56.0 [50.8, 61.3]	59.5 [56.9, 62.1]	54.2 [51.6, 56.8]	55.1 [50.9, 59.4]	51.3 [45.5, 57.1]	52.5 [47.4, 57.7]	52.0 [47.1, 56.9]	53.4 [51.0, 55.8]
A little worse off	16.2 [13.8, 18.6]	18.2 [15.5, 20.8]	18.2 [15.5, 20.8]	18.9 [15.5, 22.4]	20.1 [16.6, 23.7]	17.8 [15.8, 19.7]	18.4 [15.7, 21.1]	21.0 [17.7, 24.2]	17.5 [13.7, 21.2]	19.1 [14.8, 23.4]	18.6 [14.4, 22.8]	18.6 [16.4, 20.9]
Much worse off	9.4 [7.1, 11.6]	9.6 [7.4, 11.8]	12.7 [9.5, 15.9]	10.5 [7.0, 13.9]	15.5 [10.5, 20.6]	11.1 [8.9, 13.4]	7.7 [6.0, 9.4]	9.7 [6.5, 12.9]	14.4 [10.1, 18.7]	12.2 [9.3, 15.1]	18.8 [13.0, 24.7]	11.1 [9.0, 13.3]
Current financial situation (Feb/March 2021)												
Better off	18.6 [17.0, 20.1]	18.1 [16.1, 20.2]	16.4 [13.7, 19.0]	15.6 [13.2, 18.0]	10.4 [8.3, 12.4]	16.3 [15.1, 17.4]	24.7 [22.8, 26.6]	20.6 [17.1, 24.2]	18.9 [15.6, 22.2]	20.6 [16.9, 24.3]	13.5 [10.7, 16.3]	21.2 [19.5, 23.0]
About the same	52.1 [49.7, 54.4]	53.4 [50.1, 56.6]	48.5 [44.4, 52.6]	52.8 [48.8, 56.8]	49.3 [45.4, 53.1]	51.4 [49.4, 53.4]	49.1 [46.0, 52.2]	47.4 [43.2, 51.7]	48.8 [44.2, 53.4]	46.6 [41.5, 51.8]	48.2 [42.9, 53.4]	48.6 [45.6, 51.5]
A little worse off	18.6 [16.6, 20.7]	18.0 [15.6, 20.4]	20.0 [16.3, 23.7]	16.1 [13.4, 18.8]	21.3 [16.2, 26.3]	18.9 [16.7, 21.1]	16.6 [14.6, 18.5]	19.6 [15.3, 24.0]	19.7 [15.4, 23.9]	17.2 [13.9, 20.6]	20.2 [16.5, 23.8]	17.9 [16.1, 19.8]
Much worse off	10.7 [9.3, 12.2]	10.5 [8.4, 12.5]	15.1 [12.0, 18.3]	15.5 [12.6, 18.4]	19.0 [15.8, 22.3]	13.4 [12.1, 14.7]	9.6 [7.7, 11.5]	12.3 [9.4, 15.2]	12.6 [9.4, 15.7]	15.6 [11.9, 19.2]	18.1 [14.1, 22.1]	12.3 [10.3, 14.2]
Change in employment status (March–May 2020)												
Work–work	34.9 [31.2, 38.6]	33.0 [30.0, 36.0]	32.5 [27.0, 38.0]	32.1 [27.4, 36.6]	23.7 [18.9, 28.5]	31.8 [28.6, 34.9]	59.7 [56.6, 62.8]	52.3 [46.7, 57.9]	52.2 [46.9, 57.6]	47.0 [41.4, 52.4]	34.1 [29.2, 38.9]	52.3 [49.3, 55.2]
Work–furlough	25.2 [21.3, 29.1]	23.9 [20.3, 27.5]	23.5 [17.7, 29.4]	21.6 [17.1, 26.2]	27.2 [21.3, 33.0]	24.8 [21.2, 28.3]	25.5 [22.6, 28.4]	30.2 [24.3, 36.2]	26.7 [21.6, 31.7]	27.1 [22.0, 32.2]	25.0 [20.4, 29.6]	26.1 [23.4, 28.8]
Work–not work	3.8 [2.1, 5.5]	4.3 [2.3, 6.2]	3.0 [0.5, 5.4]	5.1 [2.3, 8.0]	5.7 [2.2, 9.1]	4.3 [2.5, 6.1]	4.3 [1.5, 7.0]	3.2 [0.9, 5.5]	6.1 [1.31, 11.0]	3.3 [0.3, 6.2]	4.7 [1.0, 8.4]	4.4 [1.8, 7.0]
Other	36.1 [32.7, 39.6]	38.8 [34.6, 43.0]	41.0 [35.8, 46.1]	41.2 [35.9, 46.4]	43.4 [37.3, 49.7]	39.1 [35.6, 42.7]	10.4 [8.4, 12.6]	14.3 [10.1, 18.4]	15.0 [11.0, 18.9]	22.7 [17.4, 27.9]	36.2 [30.4, 41.9]	17.3 [14.7, 19.8]
Change in employment status (March–Sept/Oct 2020)												
Work–work	51.0 [48.4, 53.5]	47.4 [44.3, 50.6]	47.2 [42.5, 51.9]	45.7 [42.6, 48.7]	39.2 [35.1, 43.4]	47.0 [44.9, 49.2]	79.1 [76.9, 81.2]	75.2 [71.2, 79.2]	66.8 [61.4, 72.2]	67.8 [63.4, 72.2]	50.4 [45.4, 55.6]	70.9 [68.6, 73.3]
Work–furlough	6.9 [5.0, 8.7]	6.4 [4.3, 8.6]	6.9 [4.0, 9.8]	7.0 [4.9, 9.1]	8.4 [5.0, 11.9]	7.1 [5.4, 8.8]	4.8 [3.6, 6.1]	3.9 [2.4, 5.3]	8.2 [5.1, 11.4]	5.1 [2.7, 7.4]	5.7 [2.8, 8.4]	5.3 [4.0, 6.6]
Work–not work	4.6 [3.6, 5.7]	5.8 [4.4, 7.3]	5.4 [3.3, 7.5]	5.0 [3.3, 6.7]	4.8 [2.6, 6.9]	5.0 [4.1, 5.9]	4.6 [3.3, 5.8]	4.8 [1.8, 7.7]	5.1 [2.4, 7.7]	4.4 [2.5, 6.4]	6.1 [2.5, 9.7]	4.9 [3.3, 6.5]
Other	37.5 [35.0, 40.0]	40.3 [36.9, 43.7]	40.5 [36.0, 45.0]	42.3 [38.7, 45.9]	47.6 [42.6, 52.6]	40.9 [38.4, 43.4]	11.5 [9.9, 13.1]	16.1 [13.1, 19.2]	19.9 [15.7, 24.1]	22.7 [19.0, 26.4]	37.8 [32.7, 43.0]	18.8 [16.8, 20.8]
Change in employment status (March 2020 –Feb/Mar 2021)												
Work–work	45.0 [43.3, 46.6]	44.7 [41.7, 47.6]	36.6 [33.2, 40.0]	40.9 [36.8, 45.0]	29.6 [26.4, 32.6]	40.4 [39.1, 41.8]	73.3 [70.7, 75.9]	69.4 [65.7, 73.0]	61.6 [56.6, 66.5]	59.9 [55.7, 64.1]	46.2 [41.2, 51.1]	65.4 [62.9, 67.9]
Work–furlough	11.6 [9.7, 13.4]	8.6 [7.0, 10.3]	14.7 [11.4, 18.0]	11.2 [9.0, 13.4]	16.3 [13.2, 19.4]	12.3 [11.0, 13.7]	12.1 [10.4, 13.9]	11.8 [9.2, 14.3]	15.7 [12.2, 19.2]	13.5 [10.8, 16.2]	2.5 [9.6, 15.5]	12.7 [11.3, 14.0]
Work–not work	7.8 [6.4, 9.1]	8.4 [6.9, 10.0]	9.8 [7.3, 12.2]	8.3 [6.0, 10.6]	6.6 [4.4, 8.7]	7.9 [6.9, 9.0]	3.7 [2.6, 4.7]	4.9 [2.9, 7.0]	5.2 [2.3, 8.0]	4.4 [2.3, 6.7]	5.1 [3.1, 7.2]	4.3 [3.3, 5.3]
Other	35.7 [34.0, 37.3]	38.3 [35.6, 41.0]	38.9 [35.4, 42.3]	39.6 [36.0, 43.3]	47.6 [43.4, 51.7]	39.3 [37.7, 40.8]	10.9 [9.0, 12.8]	13.9 [10.9, 17.0]	17.6 [14.0, 21.1]	22.1 [18.4, 25.9]	36.2 [32.0, 40.4]	17.7 [15.6, 19.7]
New benefit claims made since March 2020 (to Feb/March 2021)												
	18.7 [16.8, 20.6]	17.3 [14.6, 20.0]	21.5 [18.4, 24.6]	22.8 [19.5, 26.1]	25.7 [22.2, 29.2]	20.7 [19.2, 22.2]	15.9 [14.1, 17.6]	20.2 [16.8, 23.6]	20.4 [16.4, 24.4]	22.9 [19.1, 26.6]	24.6 [20.0, 29.1]	19.0 [17.0, 21.0]
Payment holidays since March 2020												
	7.3 [5.9, 8.6]	6.8 [5.2, 8.4]	10.0 [7.6, 12.3]	9.3 [7.0, 11.6]	12.4 [9.4, 15.6]	8.8 [7.5, 10.0]	15.0 [13.1, 16.9]	20.5 [16.7, 24.4]	17.5 [12.8, 22.3]	17.5 [13.8, 21.3]	20.6 [16.9, 24.4]	17.1 [15.1, 19.0]

Increased financial help from family and friends												
	2.3 [1.5, 3.1]	3.3 [2.2, 4.4]	5.1 [3.0, 7.2]	4.3 [2.5, 6.0]	7.2 [4.3, 10.1]	4.0 [2.9, 5.1]	2.0 [1.4, 2.6]	2.9 [1.8, 4.3]	3.6 [1.9, 5.3]	3.7 [2.2, 5.2]	3.7 [1.6, 5.9]	2.7 [1.8, 3.6]
Other methods for mitigating economic shock												
Spending less	18.1 [16.0, 20.2]	18.8 [16.7, 20.8]	22.3 [18.6, 25.9]	18.9 [15.7, 22.1]	22.4 [18.4, 26.4]	19.6 [17.7, 21.6]	18.0 [15.8, 20.1]	19.1 [15.8, 22.4]	22.2 [17.4, 26.9]	23.4 [19.4, 27.4]	24.9 [21.2, 28.4]	20.3 [18.3, 22.3]
Using savings	14.4 [12.7, 16.3]	13.1 [11.3, 14.9]	16.7 [12.7, 20.6]	17.5 [14.6, 20.4]	20.3 [17.2, 23.4]	16.0 [14.4, 17.5]	11.9 [10.3, 13.5]	13.9 [10.5, 17.3]	18.6 [14.3, 22.9]	16.8 [13.4, 20.3]	18.4 [15.0, 21.9]	14.4 [12.7, 16.3]
Borrowing bank or credit card	2.5 [1.8, 3.3]	2.3 [1.2, 3.4]	4.1 [2.0, 6.1]	3.5 [1.9, 5.1]	6.4 [4.3, 8.4]	3.6 [2.8, 4.4]	4.5 [3.5, 5.6]	7.2 [4.8, 9.7]	8.1 [4.7, 11.4]	4.6 [2.7, 6.4]	10.2 [6.2, 14.3]	6.3 [4.8, 7.7]
Borrowing friends and family	1.5 [0.8, 2.2]	1.5 [0.5, 2.6]	3.1 [1.3, 4.9]	2.7 [0.8, 4.6]	3.9 [1.6, 6.3]	2.3 [1.4, 3.3]	3.3 [2.2, 4.4]	4.9 [1.7, 8.2]	3.8 [1.6, 5.9]	6.6 [3.6, 9.7]	13.4 [8.8, 18.1]	5.7 [4.1, 7.4]

On Universal Credit (January 2020-March 2021)												
	7.3 [5.9, 8.8]	5.9 [4.0, 7.7]	12.3 [9.4, 15.2]	11.4 [8.5, 14.4]	19.3 [15.5, 23.1]	10.5 [8.9, 12.1]	6.8 [5.5, 8.1]	9.6 [6.7, 12.5]	14.7 [11.3, 18.1]	11.0 [7.2, 14.9]	18.4 [13.6, 23.4]	10.5 [8.6, 12.4]

At the beginning of the pandemic, a quarter of participants in the NCDS (24.8%, 95% CI 21.2, 28.3) and BCS70 (26.1%, 95% CI 23.4, 28.8) were put on furlough, by early autumn, this had reduced to 7.1% (95% CI 5.4, 8.8) and 5.3% (95% CI 4.0, 6.6) for the NCDS and BCS70, respectively, and increased moderately in the spring of 2021, while a few had lost their jobs. For those working, there was no difference in the proportion who were furloughed or lost their jobs during the pandemic by distinct psychological distress trajectories.

Overall, 20.7% (95% CI 19.2, 22.2) of the NCDS and 19% (95% CI 17.0, 21.0) of the BCS70 made new benefit claims, and 17.1% (95% CI 15.1, 19.0) of the BCS70 and 8.8% (95% CI 7.5, 10.0) of the NCDS took payment holidays. In the NCDS, a higher proportion of the ‘stable-high’ trajectory made new benefits claims (25.7%, 95% CI 22.2, 29.2) and took payment holidays (12.4%, 95% CI 9.4, 15.6) compared to the ‘no symptom’ (18.7%, 95% CI 16.8, 20.6; 7.3%, 95% CI 5.9, 8.6) and ‘low-symptom’ (17.3%, 95% CI 14.6, 20.0; 6.8%, 95% CI 5.2, 8.4) groups. While in the BCS70, the ‘stable-high’ (24.6%, 95% CI 20.0, 29.1) and ‘midlife-onset’ (22.9%, 95% CI 19.1, 26.6) trajectory groups made new benefit claims compared to the ‘no-symptoms’ (15.9%, 95% CI 14.1, 17.6) trajectory.

A higher proportion of cohort members reduced consumption or used savings during the pandemic to mitigate the financial shock, while a smaller proportion borrowed from family or formal institutions. However, compared to the ‘no-symptoms’ trajectory (NCDS: 2.5%, 95% CI 1.8,3.3; BCS70: 4.5%, 95% CI 3.5,5.6), a higher proportion of the ‘stable-high’ trajectories in both cohorts borrowed from banks or credit cards (NCDS: 6.4%, 95% CI 4.3, 8.4; BCS70: 10.2%, 95% CI 6.2, 14.3), and in the BCS70 borrowed from family and friends (13.4%, 95% CI 8.8, 18.1) than the ‘no-symptoms’, ‘early-adult onset’, and ‘adult-onset’ groups.

### Risk of change in economic outcomes during the course of the pandemic

Table 2 presents the relative risks in the fully adjusted models, associated with each of the economic outcomes during the pandemic for different trajectories of psychological distress, with the largest trajectory ‘no symptoms’ used as the reference category in the analysis.

**Table 2:** Relative risk (RR) of change in financial and employment circumstances associated with pre-pandemic psychological distress trajectories in the NCDS and BCS70 during the COVID-19 pandemic

Outcomes during COVID Ref: No symptoms	NCDS (*n* = 15,291)				BCS70 (*n* = 16,128)			
	Stable low	Adult onset	Midlife onset	Stable high	Early-adult onset	Adult onset	Midlife onset	Stable high
Current financial situation (May 2020)								
Better off	1.00 [0.78, 1.28]	1.00 [0.74, 1.35]	0.93 [0.69, 1.25]	0.73 [0.52, 1.03]	0.87 [0.67, 1.13]	1.24 [0.96, 1.44]	1.07 [0.80, 1.44]	1.00 [0.74, 1.37]
A little worse off	1.06 [0.90, 1.25]	1.04 [0.79, 1.37]	1.10 [0.87, 1.37]	1.09 [0.88, 1.35]	0.79 [0.59, 1.06]	1.34 [0.95, 1.89]	1.24 [0.93, 1.64]	1.39 [1.03, 1.89]
Much worse off	1.22 [0.93, 1.59]	1.66 [1.21, 2.45]	1.17 [0.78, 1.75]	1.73 [1.09, 2.75]	1.17 [0.84, 1.64]	1.29 [0.82, 2.02]	1.18 [0.82, 1.70]	1.89 [1.29, 2.77]
Current financial situation (Sep/Oct 2020)								
Better off	0.88 [0.72, 1.08]	0.90 [0.67, 1.20]	0.79 [0.62, 1.01]	0.83 [0.65, 1.06]	0.77 [0.62, 0.95]	0.99 [0.74, 1.34]	0.91 [0.69, 1.20]	0.72 [0.56, 0.93]
A little worse off	1.20 [0.99, 1.45]	1.21 [0.92, 1.59]	1.23 [0.96, 1.58]	1.54 [1.17, 2.03]	1.13 [0.88, 1.46]	1.02 [0.74, 1.39]	1.07 [0.77, 1.49]	1.10 [0.82, 1.46]
Much worse off	1.15 [0.85, 1.57]	1.48 [1.04, 2.10]	1.16 [0.81, 1.66]	1.93 [1.31, 2.84]	1.25 [0.86, 1.81]	1.99 [1.30, 3.05]	1.70 [1.17, 2.46]	2.48 [1.71, 3.59]
Current financial situation (Feb/March 2021)								
Better off	0.96 [0.82, 1.13]	1.09 [0.85, 1.39]	0.89 [0.73, 1.09]	0.75 [0.60, 0.92]	0.96 [0.78, 1.17]	0.87 [0.69, 1.10]	0.96 [0.76, 1.22]	0.78 [0.59, 1.02]
A little worse off	0.98 [0.83, 1.15]	1.20 [0.95, 1.51]	0.89 [0.70, 1.13]	1.27 [1.01, 1.60]	1.28 [0.96, 1.70]	1.23 [0.92, 1.64]	1.11 [0.87, 1.41]	1.31 [1.03, 1.66]
Much worse off	1.02 [0.78, 1.32]	1.60 [1.20, 2.14]	1.50 [1.15, 1.96]	2.10 [1.57, 2.81]	1.34 [1.00, 1.79]	1.36 [1.00, 1.85]	1.77 [1.29, 2.43]	2.08 [1.62, 2.65]
Change in employment status (March–May 2020)								
Work–furlough	1.22 [0.93, 1.59]	1.66 [1.12, 2.45]	1.17 [0.78, 1.75]	1.73 [1.09, 2.75]	1.24 [0.92, 1.67]	1.05 [0.83, 1.33]	1.27 [0.98, 1.66]	1.31 [0.99, 1.73]
Work–not work	1.06 [0.90, 1.25]	1.04 [0.79, 1.37]	1.10 [0.87, 1.38]	1.09 [0.88, 1.35]	0.73 [0.33, 1.62]	1.45 [0.66, 3.17]	0.89 [0.35, 2.28]	1.39 [0.65, 2.94]
Change in employment status (March–Sept/Oct 2020)								
Work–furlough	1.05 [0.75, 1.48]	1.09 [0.67, 1.77]	1.14 [0.82, 1.58]	1.64 [1.09, 2.47]	0.84 [0.54, 1.32]	2.02 [1.28, 3.21]	1.22 [0.75, 1.97]	1.81 [1.13, 2.91]
Work–not work	1.38 [0.96, 1.99]	1.35 [0.85, 2.13]	1.23 [0.88, 1.71]	1.49 [0.89, 2.49]	0.99 [0.57, 1.74]	1.25 [0.79, 1.97]	1.12 [0.69, 1.81]	1.73 [1.02, 2.93]
Change in employment status (March 2020–Feb/Mar 2021)								
Work–furlough	0.75 [0.56, 0.99]	1.45 [1.09, 1.93]	1.03 [0.78, 1.37]	1.95 [1.45, 2.63]	0.88 [0.65, 1.17]	1.36 [1.02, 1.81]	1.21 [0.89, 1.65]	1.20 [0.87, 1.64]
Work–not work	1.07 [0.85, 1.35]	1.55 [1.17, 2.03]	1.14 [0.83, 1.57]	1.27 [0.85, 1.91]	1.32 [0.76, 2.29]	1.61 [0.85, 2.45]	1.44 [0.85, 2.45]	2.00 [1.18, 3.38]

Parameters are adjusted for sex, breastfed, mother smoked during pregnancy, gestation period, birthweight, parental social class at 0, parental education at 0, parental income, housing tenure at 7/5, access to house amenities at 7/5, total household income at 0, crowding at age 0, 7/5 and 11/10, parents marital status at 0, maternal age at birth, mother worked in first five years, separated from child for more than a month < age 5, read to at 7/5, CM wet the bed at 7/5, had any medical conditions at 7/5, Body Mass Index (BMI) at 11/10, and cognitive ability at 7/5 and 11/10

*RRR* Relative Risk ratio, *95% CIs* 95% confidence intervals

#### Change in financial circumstances during the course of the pandemic

Throughout the pandemic, the ‘stable-high’ trajectory group in both the NCDS (RRR = 1.7–2.1, 95% CI 1.1–1.6, 2.7–2.8) and the BCS70 (RRR = 1.9–2.5, 95% CI 1.3–1.7, 2.6–3.6), and the ‘adult-onset’ group (RRR = 1.5–1.7, 95% CI 1.0–1.2, 2.1–2.5) in the NCDS were associated with a worsening financial situation throughout the pandemic. In the early autumn 2020 and spring of 2021 the BCS70 ‘adult-onset’ (RRR = 1.4–2.0, 95% CI 1.0–1.3, 1.9–3.1) and ‘midlife-onset’ (RRR = 1.7–1.8, 95% CI 1.2–1.3, 2.4–2.5) groups, and by spring 2021 the ‘midlife-onset’ group in the NCDS (RRR = 1.5, 95% CI = 1.2, 2.0) were also associated with a greater risk of being financially worse off. In absolute terms, between 1 in 7 and 1 in 5 of the trajectories with prior psychological distress experienced worsening financial circumstances, compared to around a tenth of the ‘no-symptoms’ trajectory group (details are available in supplementary Table S5).

#### Change in employment during the course of the pandemic

During the pandemic, if in work prior to the COVID-19 outbreak, the ‘adult-onset’ (NCDS: W1 RRR 1.7, 95% CI 1.1, 2.5, W3 RRR 1.5, 95% CI 1.1, 1.9; BCS70: W2 RRR 2.0, 95% CI 1.3, 3.2, W3 RRR 1.4 95% CI 1.0, 1.8) and ‘stable-high’ (NCDS: W1 RRR 1.7, 95% CI 1.1, 2.8, W2 RRR 1.6, 95% CI 1.1, 2.5, W3 RRR 2.0, 95% CI 1.5, 2.6; BCS70: W2 RRR 1.8, 95% CI 1.1, 2.9) trajectory groups were related to a greater risk of furlough. And by spring 2021, the ‘adult-onset’ group in the NCDS, and the ‘stable-high’ group in the BCS70, were also associated with a 55% (RRR 1.6, 95% CI 1.2, 2.0) and 100% (RRR 2.0, 95% CI 1.2, 3.4) greater risk of unemployment, respectively.

### Government support and other approaches to mitigate the economic shock

The ‘stable-high’ trajectory group (Table 3) was associated with around a 43% (RR 1.43 95% CI 1.2,1.7) and 31% (RR 1.31 95% CI 1.1,1.6) increase in the risk of making new benefit claims in the BCS70 and NCDS, respectively. Likewise, the ‘midlife-onset’ trajectory group were more likely to access government support (NCDS: RR 1.21, 95% CI 1.0, 1.4; BCS70: RR 1.41, 95% CI 1.2, 1.7). Also, the ‘stable-high’ trajectory group had a 72% (RR 1.72, 95% CI 1.3, 2.3) in the NCDS and 30% (RR 1.30, 95% CI 1.1, 1.6) in the BCS70 greater risk of taking payment holidays during the pandemic (in absolute terms this represented 12.4% (95% CI 10.4, 14.8) in the NCDS and 19.1% (95% CI 16.1, 22.6) in the BCS70 of the ‘stable-high’ trajectory—see supplementary Table S6).

**Table 3:** Relative risk (RR) of using methods to mitigate the economic shock associated with pre-pandemic psychological distress trajectories in the NCDS and BCS70 during the COVID-19 pandemic

Outcomes during COVID Ref: No symptoms	NCDS (*n* = 15,291)				BCS70 (*n* = 16,128)			
	Stable low	Adult onset	Midlife onset	Stable high	Early-adult onset	Adult onset	Midlife onset	Stable high
Any new benefit claims March 2020–Feb/March 2021								
	0.95 [0.81, 1.12]	1.11 [0.92, 1.35]	1.21 [1.02, 1.42]	1.31 [1.11, 1.55]	1.21 [1.02, 1.43]	1.25 [1.04, 1.50]	1.41 [1.20, 1.66]	1.43 [1.23, 1.67]
Payment holidays March 2020–Feb/March 2021								
	0.98 [0.77, 1.26]	1.37 [1.03, 1.82]	1.29 [0.99, 1.69]	1.72 [1.31, 2.25]	1.34 [1.10, 1.64]	1.13 [0.88, 1.44]	1.16 [0.92, 1.46]	1.30 [1.08, 1.57]
Increased financial help from family and friends								
	1.45 [0.98, 2.15]	2.05 [1.36, 3.10]	1.80 [1.06, 2.80]	2.85 [1.87, 4.35]	1.34 [0.83, 2.17]	1.79 [1.21, 2.63]	1.73 [1.19, 2.51]	1.73 [1.18, 2.54]
Other methods for mitigating economic shock								
Spending less	1.07 [0.93, 1.24]	1.27 [1.06, 1.53]	1.06 [0.97, 1.24]	1.31 [1.12, 1.53]	1.07 [0.87, 1.31]	1.25 [1.00, 1.57]	1.32 [1.08, 1.61]	1.41 [1.21, 1.62]
Using savings	0.93 [0.78, 1.12]	1.17 [0.94, 1.45]	1.23 [1.01, 1.51]	1.46 [1.25, 1.70]	1.22 [0.96, 1.55]	1.59 [1.30, 1.95]	1.45 [1.16, 1.82]	1.60 [1.33, 1.92]
Borrowing from bank or using credit card	0.97 [0.59, 1.59]	1.62 [0.93, 2.82]	1.41 [0.78, 2.55]	2.59 [1.74, 3.83]	1.57 [1.04, 2.37]	1.66 [1.10, 2.52]	0.96 [0.62, 1.50]	1.88 [1.27, 2.77]
Borrowing from friends and family	0.97 [0.44, 2.15]	1.98 [0.97, 4.03]	1.62 [0.82, 3.22]	2.44 [1.37, 4.35]	1.37 [0.80, 2.34]	1.00 [0.54, 1.85]	1.84 [1.12, 3.04]	2.93 [2.02, 4.24]
On Universal Credit (Jan 2020-March 2021)								
	0.83 [0.61, 1.23]	1.47 [1.18, 1.84]	1.45 [1.12, 1.88]	2.10 [1.65, 2.66]	1.20 [0.93, 1.56]	1.74 [1.39, 2.16]	1.38 [1.03, 1.85]	1.65 [1.31, 2.08]

Parameters are adjusted for sex, breastfed, mother smoked during pregnancy, gestation period, birthweight, parental social class at 0, parental education at 0, parental income, housing tenure at 7/5, access to house amenities at 7/5, total household income at 0, crowding at age 0, 7/5 and 11/10, parents marital status at 0, maternal age at birth, mother worked in first five years, separated from child for more than a month < age 5, read to at 7/5, CM wet the bed at 7/5, had any medical conditions at 7/5, Body Mass Index (BMI) at 11/10, and cognitive ability at 7/5 and 11/10

*RR* Relative Risk, *95% CIs* 95% confidence intervals

The ‘stable-high’ trajectory group was also at a higher risk of using a variety of private methods to mitigate the economic shock, including reducing consumption, using savings, receiving financial help from family and borrowing. In particular, the NCDS ‘stable-high’ trajectory group were associated with a 2.8 fold (RR 2.85, 95% CI 1.9, 4.4) risk of relying on financial help from relatives, as well as 2.6 times (RR 2.59, 95% CI 1.7, 3.8) the risk of borrowing from a bank or using credit cards. Similarly, the ‘stable-high’ trajectory group in the BCS70 was associated with a 2.9- (RR 2.93, 95% CI 2.0, 4.2) and 1.9- (RR 1.88, 95% CI 1.3, 2.8) fold risk of borrowing, from family and financial institutions, respectively.

In the NCDS, the ‘adult-onset’ trajectory group was associated with a 105% (RR 2.05, 95% CI 1.4, 3.1) increase in receiving financial help from family, and a 37% (RR 1.37, 95% CI 1.0, 1.8) increase of taking payment holidays, and a 27% (RR 1.27, 95% CI 1.1, 1.5) increase in risk of a reduction in spending; while the ‘midlife-onset’ trajectory group were at a 80% (RR 1.80, 95% CI 1.1, 2.8) increased risk of relying on family financial support and a 23% (RR 1.23, 95% CI 1.0, 1.5) increased risk of using their savings.

In the BCS70, the ‘adult-onset’ and ‘midlife-onset’ trajectory groups were more likely to have used their savings and received financial support from family. In addition, the ‘midlife onset’ trajectory reduced consumption. The ‘adult-onset’ and ‘early-adult onset’ trajectory groups were also associated with a 66% (RR 1.66, 95% CI 1.1, 2.5) and 57% (RR 1.57, 95% CI 1.0, 2.4) increase in risk of borrowing from banks and credit cards, respectively. Within each of the trajectories, a higher proportion were making new benefit claims, reducing consumption, using savings, and taking payment holidays to mitigate the economic shock, a lower proportion were borrowing or increasing financial support from friends (See Table S6).

From January 2020 to May 2021, as shown in Table 3, the ‘adult-onset’, midlife-onset’ and ‘stable-high’ trajectory groups were more likely to be claiming Universal Credit and therefore more at risk of impact from the loss of the temporary weekly £20 Universal Credit uplift available during the pandemic.

## Discussion

While the COVID-19 economic shock affected labor market outcomes and financial circumstances for many adults, this study found the impact was disproportionately borne from those with pre-pandemic life-course psychological distress trajectories. For those in employment pre-pandemic, in both cohorts, two trajectory groups, the ‘adult-onset’ (early 30s) and the ‘stable-high’ were related to changes in their employment situations. Although supported by the JRS scheme, by March 2021, the ‘stable-high’ group in the BCS70 and ‘adult-onset’ group in the NCDS were also associated with a greater relative risk of unemployment. During COVID-19, the ‘stable-high’ and ’midlife-onset’ trajectory groups were also supported by the benefits system. Despite the government response, trajectories with pre-pandemic psychological distress were also associated with adapting their financial behavior, including reduced consumption, dis-saving, and relying on increased financial help from family and friends to mitigate worsening financial circumstances. Notably, the ‘stable-high’ trajectory group, along with a greater risk of borrowing from banks or credit cards for the ‘early-adult onset’ and ‘adult-onset’ trajectory groups in the BCS70, were associated with alleviating their financial circumstances by taking payment holidays and borrowing from institutions and friends, which longer-term if mismanaged may lead to financial difficulties.

As with other studies, we find that individuals were differentially exposed to the economic impact of COVID in the UK [7–9] and specifically individuals with poor pre-pandemic mental health [10]. Here the ‘adult-onset’ in the NCDS and ‘stable-high’ symptom groups in the BCS70 were related to a greater relative risk of unemployment. Multiple mechanisms (beyond the scope of this work) might explain why these two life-course psychological distress trajectories were associated with employment changes during the pandemic. For example, both trajectories were related to higher levels of psychological distress in their early thirties, which may coincide with an important period of employment transition [51], thus reducing human capital accumulation and skill acquisition [15] which in turn influences future employment outcomes by maintaining gainful employment and/or reducing access to society’s opportunity structures. During COVID-19, the labor market shock was heterogenous, industries which involved contact with people and ‘elementary’ occupations were hardest hit [8] as well as low earners [9]. Working in these sectors may be as a consequence of historically sensitive periods in career development. Also, studies suggest there is evidence of discrimination against applicants with a history of mental health problems [52] and a greater impact of job loss during recessions [5]. For example, for the NCDS cohort their early thirties (‘adult-onset’) coincided with the 1990–93 recession, where unemployment rates increased to over 10%, which may have resulted in poorer mental health, or their poorer mental health may have resulted in less favorable employment opportunities.

At an aggregate level, the economic policy measures taken by the government, especially the JRS and UC uplift, were successful in broadly insuring households against the economic shock [53]. Indeed, in this study during the pandemic, differential psychological distress trajectories with symptoms pre-pandemic were increasingly likely to have been supported by the JRS and benefit system. However, despite this support, most trajectories with pre-pandemic psychological distress were associated with a relative risk of worsening financial circumstances. The coronavirus JRS ceased from the 30 September 2021, and the £20 UC uplift concluded on the 6 October 2021. Removal of this support could be particularly detrimental for those with pre-pandemic psychological distress. In addition, this loss of financial support will be compounded by increases in the cost of living, rising National Insurance Contributions in April 2022, and future economic uncertainty over COVID-19, Brexit, the national debt, and climate change, which may further exacerbate economic inequalities for those with pre-pandemic psychological distress. Groups with pre-pandemic psychological distress may be less resilient and more susceptible to the negative effects of the current cost of living crisis.

In this study, psychological distress trajectory groupings with prior symptoms were associated with greater relative use of different mitigation strategies, including appropriate adaptive methods such as dis-saving, and reduced consumption. Though, particularly worrying was the association between the ‘stable-high’ symptoms trajectory in both cohorts and in the BCS70 the ‘early-adult onset’ and ‘adult-onset’ trajectory groups with using methods such as payment holidays, and borrowing from friends and institutions, albeit on aggregate within trajectory the least likely approaches. Perhaps, the use of these strategies were needed because of fewer economic resources accumulated for individuals with prior mental health problems, and in particular for the younger BCS70 cohort. If borrowing and payment holidays are not short-term solutions to the COVID-19 economic crisis, they could lead to problem debt. A meta-analysis of pooled odds ratios showed a significant relationship between debt and mental disorder (OR = 3.24) and depression (OR = 2.77). [54]. In prior studies indebtedness or an increase in debt levels was associated with subsequently poorer mental [55], and more severe the debt being related to more severe health difficulties [54]. Previous work has also shown the relationship between low income and mental health is largely mediated by debt [56, 57].

The relationship between mental health and economic hardship is complex and contentious, whether the relationship is better explained by social causation, social selection or both [15, 58]. In this study, we investigated whether the consequences of life-course psychological distress trajectories after an unprecedented economic shock were related to poorer financial and employment outcomes. Although this was an abrupt event, we cannot rule out the influence of prior financial circumstances, both as a confounder and as an aggravation of economic inequalities for those with prior poor mental health. We conducted further analysis (Tables S7–S9) to examine how trajectory groups with prior psychological distress were managing financially prior to the COVID-19 pandemic. Compared to the ‘no-symptoms’ trajectory, none were associated with living comfortably, and the trajectory groups with higher proximal symptoms of psychological distress were at a greater relative risk of financial difficulties pre-pandemic. We stratified the samples into financially comfortable and financially struggling pre-pandemic and found that the risk of being worse off during COVID-19 for the ‘stable-high’ trajectory groups was associated with both those struggling, as well those who were financially comfortable pre-pandemic. In addition, psychological distress trajectory groups with onset at different stages in the life-course (‘adult-onset’ and ‘midlife-onset’) who were comfortable pre-pandemic were at risk of a worsening financial situation. This indicates both a possible increase in economic inequalities as a result of the pandemic, as well as a likely vulnerability for some adult life-course psychological distress trajectory groups to economic shocks.

### Strengths and limitations

Our study relates long-term individual longitudinal psychological distress data before the COVID-19 pandemic to economic outcomes during the COVID-19 pandemic. Other unique strengths include nationally representative samples, large sample sizes, prospective follow-up from birth to midlife, and economic data collected at three discrete time-points during the COVID-19 pandemic from May 2020 to March 2021.

There are a number of limitations of this study. Our findings can only be generalized to those born in Britain in 1958 and 1970 or close to those years. As with most longitudinal research, selective attrition has occurred. However, we included auxiliary variables in the multiple imputation models, including mental health and related variables from birth, which allows for predicting missing data with greater accuracy and minimizing non-random variation in these values [59]. Also bias due to unmeasured confounding especially time varying confounding, in the observed association between the pre-pandemic psychological distress trajectories and economic outcomes during the pandemic cannot be ruled out. Although, extensive early life factors were accounted for, there is no guarantee we have sufficiently controlled for all relevant confounding factors. In terms of the method adopted, latent classes are approximations of symptom patterns in the data and do not represent actual data points, but are evidenced based summaries of psychological distress in the cohorts (see Table S3a–3c for more limitations on this approach). Also, relationships between subjective measures of poor financial situation and depression can arise irrespective of ‘objective’ measures of financial situation, therefore indicating a person-specific effect [60]. However, in this study, the measures of psychological distress and economic outcomes were time variant, and we investigated both subjective measures of financial circumstance and ‘objective’ measures of employment situation and mitigation methods during COVID-19.

## Conclusions

Economic inequalities for pre-pandemic psychological distress trajectories with differential onset, severity and chronicity across the life-course seem to have been exacerbated by the COVID-19 economic shock. Groupings with poor mental health earlier in the life-course, as well as in midlife may be vulnerable to economic shocks. During the pandemic, life-course psychological distress trajectory groups with prior symptoms were more likely to seek support from the governments’ economic response package. However, the subsequent cut in support, alongside further ‘post-pandemic’ economic challenges will put further strain on the finances of those that have experienced psychological distress over their life-course. In addition, for some groups personal adaptations in financial behavior to mitigate the economic shock during the COVID-19 pandemic may not be sustainable, including reduced consumption during a period of increasing inflation, while indefinitely relying on savings, and financial support networks, which may themselves be fragile. The bidirectional association of mental health and financial difficulties could in turn increase psychological distress symptoms or reduce the chances and delay improvements in current and future mental health. Highlighting the different mental health trajectory groups across the life-course which are vulnerable to economic shock and likely in need of further financial and mental health support is crucial. More research is needed to explore the possible mechanisms throughout the adult life-course related to vulnerability to labor market and economic shocks, as well as monitoring these trajectories over the short and medium term, especially in relation to employment opportunities, debt problems, and increased mental health symptomatology.

## Supplementary Information

Below is the link to the electronic supplementary material.

Supplementary file1 (DOCX 133 KB)

## Data Availability

The data are freely available from the UK Data Service at https://ukdataservice.ac.uk/.

## References

[1] Holmes EA, O'Connor RC, Perry VH, Tracey I, Wessely S, Arseneault L, Ballard C, Christensen H, Silver RC, Everall I, Ford T (2020). Multidisciplinary research priorities for the COVID-19 pandemic: a call for action for mental health science. Lancet Psychiatry.

[2] Douglas M, Katikireddi SV, Taulbut M, McKee M, McCartney G (2020). Mitigating the wider health effects of covid-19 pandemic response. BMJ.

[3] Ahmed F, Ahmed NE, Pissarides C, Stiglitz J (2020). Why inequality could spread COVID-19. Lancet Public Health.

[4] Egan M, Daly M, Delaney L (2015). Childhood psychological distress and youth unemployment: evidence from two British cohort studies. Soc Sci Med.

[5] Evans-Lacko S, Knapp M, McCrone P, Thornicroft G, Mojtabai R (2013). The mental health consequences of the recession: economic hardship and employment of people with mental health problems in 27 European countries. PLoS ONE.

[6] Egan M, Daly M, Delaney L (2016). Adolescent psychological distress, unemployment, and the great recession: evidence from the National Longitudinal Study of Youth 1997. Soc Sci Med.

[7] Crossley TF, Fisher P, Low H (2021). The heterogeneous and regressive consequences of COVID-19: evidence from high quality panel data. J Public Econ.

[8] Benzeval M, Burton J, Crossley TF, Fisher P, Jäckle A, Low H, Read B (2020). The idiosyncratic impact of an aggregate shock: the distributional consequences of COVID-19. SSRN.

[9] Brewer M, Gardiner L (2020). The initial impact of COVID-19 and policy responses on household incomes. Oxf Rev Econ Policy.

[10] Di Gessa G, Maddock J, Green MJ, Thompson EJ, McElroy E, Davies HL (2022). Pre-pandemic mental health and disruptions to healthcare, economic and housing outcomes during the COVID-19 pandemic: evidence from 12 UK longitudinal studies. Br J Psychiatry.

[11] Butterworth P, Rodgers B, Windsor TD (2009). Financial hardship, socio-economic position and depression: results from the PATH through life survey. Soc Sci Med.

[12] Kiely KM, Leach LS, Olesen SC, Butterworth P (2015). How financial hardship is associated with the onset of mental health problems over time. Soc Psychiatry Psychiatr Epidemiol.

[13] McAlpine DD, Alang SM (2021). Employment and economic outcomes of persons with mental illness and disability: the impact of the great recession in the United States. Psychiatr Rehabil J.

[14] Kröger H, Pakpahan E, Hoffmann R (2015). What causes health inequality? A systematic review on the relative importance of social causation and health selection. Eur J Public Health.

[15] Ridley M, Rao G, Schilbach F, Patel V (2020). Poverty, depression, and anxiety: causal evidence and mechanisms. Science.

[16] Lund C, Cois A (2018). Simultaneous social causation and social drift: longitudinal analysis of depression and poverty in South Africa. J Affect Disord.

[17] Reiss F (2013). Socioeconomic inequalities and mental health problems in children and adolescents: a systematic review. Soc Sci Med.

[18] Olesen SC, Butterworth P, Leach LS, Kelaher M, Pirkis J (2013). Mental health affects future employment as job loss affects mental health: findings from a longitudinal population study. BMC Psychiatry.

[19] Goodman A, Joyce R, Smith JP (2011). The long shadow cast by childhood physical and mental problems on adult life. Proc Natl Acad Sci.

[20] Smith JP, Smith GC (2010). Long-term economic costs of psychological problems during childhood. Soc Sci Med.

[21] Mousteri V, Daly M, Delaney L, Tynelius P, Rasmussen F (2019). Adolescent mental health and unemployment over the lifespan: population evidence from Sweden. Soc Sci Med.

[22] Evensen M, Lyngstad TH, Melkevik O, Reneflot A, Mykletun A (2017). Adolescent mental health and earnings inequalities in adulthood: evidence from the Young-HUNT Study. J Epidemiol Commun Health.

[23] Mojtabai R, Stuart EA, Hwang I, Susukida R, Eaton WW, Sampson N, Kessler RC (2015). Long-term effects of mental disorders on employment in the National Comorbidity Survey ten-year follow-up. Soc Psychiatry Psychiatr Epidemiol.

[24] Kessler RC, Berglund P, Demler O, Jin R, Merikangas KR, Walters EE (2005). Lifetime prevalence and age-of-onset distributions of DSM-IV disorders in the National Comorbidity Survey Replication. Arch Gen Psychiatry.

[25] Moffitt TE, Caspi A, Taylor A, Kokaua J, Milne BJ, Polanczyk G, Poulton R (2010). How common are common mental disorders? Evidence that lifetime prevalence rates are doubled by prospective versus retrospective ascertainment. Psychol Med.

[26] Colman I, Ploubidis GB, Wadsworth MEJ, Jones PB, Croudace TJ (2007). A longitudinal typology of symptoms of depression and anxiety over the life course. Biol Psychiat.

[27] Musliner KL, Munk-Olsen T, Eaton WW, Zandi PP (2016). Heterogeneity in long-term trajectories of depressive symptoms: patterns, predictors and outcomes. J Affect Disord.

[28] Paksarian D, Cui L, Angst J, Ajdacic-Gross V, Rössler W, Merikangas KR (2016). Latent trajectories of common mental health disorder risk across 3 decades of adulthood in a population-based cohort. JAMA Psychiat.

[29] Kessler RC, Amminger GP, Aguilar-Gaxiola S, Alonso J, Lee S, Ustun TB (2007). Age of onset of mental disorders: a review of recent literature. Curr Opin Psychiatry.

[30] Berndt ER, Koran LM, Finkelstein SN, Gelenberg AJ, Kornstein SG, Miller IM, Thase ME, Trapp GA, Keller MB (2000). Lost human capital from early-onset chronic depression. Am J Psychiatry.

[31] Sharac J, Mccrone P, Clement S, Thornicroft G (2010). The economic impact of mental health stigma and discrimination: a systematic review. Epidemiol Psychiatr Sci.

[32] Abramson LY, Seligman ME, Teasdale JD (1978). Learned helplessness in humans: critique and reformulation. J Abnorm Psychol.

[33] Beck AT (1979). Cognitive therapy of depression.

[34] Hills J, Bastagli F, Cowell FA, Glennerster H, Karagiannaki E, McKnight A (2013). Wealth in the UK: distribution, accumulation, and policy.

[35] Sullivan A, Brown M, Bann D (2015). Guest Editorial: generation X enters middle age. Longitud Life Course Stud.

[36] Power C, Elliott J (2006). Cohort profile: 1958 British birth cohort (national child development study). Int J Epidemiol.

[37] Mostafa T, Narayanan M, Pongiglione B, Dodgeon B, Goodman A, Silverwood RJ, Ploubidis GB (2021). Missing at random assumption made more plausible: evidence from the 1958 British birth cohort. J Clin Epidemiol.

[38] Elliott J, Shepherd P (2006). Cohort profile: 1970 British birth cohort (BCS70). Int J Epidemiol.

[39] Sullivan A, Brown M, Hamer M, Ploubidis GB (2022). Cohort profile update: the 1970 British Cohort Study (BCS70). Int J Epidemiol.

[40] Brown M, Goodman A, Peters A, Ploubidis G, Sanchez A, Silverwood R, Smith K (2001). COVID-19 survey in five national longitudinal studies: waves 1, 2 and 3: user guide (Version 3).

[41] Little RJ, Rubin DB (2019). Statistical analysis with missing data.

[42] Silverwood R, Narayanan M, Dodgeon B, Ploubidis G (2020). Handling missing data in the National Child Development Study: user guide.

[43] Von Hippel PT (2007). Regression with missing Ys: an improved strategy for analyzing multiply imputed data. Sociol Methodol.

[44] Rodgers B, Pickles A, Power C, Collishaw S, Maughan B (1999). Validity of the Malaise inventory in general population samples. Soc Psychiatry Psychiatr Epidemiol.

[45] Rutter M, Tizard J, Whitmore K (1970). Education, health and behaviour: psychological and medical study of childhood development.

[46] Drapeau A, Marchand A, Beaulieu-Prévost D, Lbate L (2012). Epidemiology of psychological distress. Mental illnesses: understanding prediction, and control.

[47] McGee R, Williams S, Silva PA (1986). An evaluation of the Malaise inventory. J Psychosom Res.

[48] Ploubidis GB, McElroy E, Moreira HC (2019). A longitudinal examination of the measurement equivalence of mental health assessments in two British birth cohorts. Longitud Life Course Stud.

[49] Furnham A, Cheng H (2015). The stability and change of malaise scores over 27 years: findings from a nationally representative sample. Personal Individ Differ.

[50] Pang M, Kaufman JS, Platt RW (2016). Studying noncollapsibility of the odds ratio with marginal structural and logistic regression models. Stat Methods Med Res.

[51] Zacher H, Froidevaux A (2021). Life stage, lifespan, and life course perspectives on vocational behavior and development: a theoretical framework, review, and research agenda. J Vocat Behav.

[52] Bjørnshagen V (2021). The mark of mental health problems A field experiment on hiring discrimination before and during COVID-19. Soc Sci Med.

[53] Bell T, Brewer M (2021) The 12-month stretch: Where the Government has delivered – and where it has failed – during the Covid-19 crisis, Resolution Foundation, March 2021. https://www.resolutionfoundation.org/app/uploads/2021/03/The-12-month-stretch.pdf/downloaded. Accessed 01 Oct 2021

[54] Richardson T, Elliott P, Roberts R (2013). The relationship between personal unsecured debt and mental and physical health: a systematic review and meta-analysis. Clin Psychol Rev.

[55] Fitch C, Hamilton S, Bassett P, Davey R (2011). The relationship between personal debt and mental health: a systematic review. Ment Health Rev J.

[56] Arber S, Fenn K, Meadows R (2014). Subjective financial well-being, income and health inequalities in mid and later life in Britain. Soc Sci Med.

[57] Jenkins R, Bhugra D, Bebbington P, Brugha T, Farrell M, Coid J, Fryers T, Weich S, Singleton N, Meltzer H (2008). Debt, income and mental disorder in the general population. Psychol Med.

[58] Bierman A, Upenieks L, Glavin P, Schieman S (2021). Accumulation of economic hardship and health during the COVID-19 pandemic: social causation or selection?. Soc Sci Med.

[59] Sterne JA, White IR, Carlin JB, Spratt M, Royston P, Kenward MG, Wood AM, Carpenter JR (2009). Multiple imputation for missing data in epidemiological and clinical research: potential and pitfalls. BMJ.

[60] Bridges S, Disney R (2010). Debt and depression. J Health Econ.

