# Supplementary material for: Adult life-course trajectories of psychological distress and economic outcomes in midlife during the COVID-19 pandemic: evidence from the 1958 and 1970 British birth cohorts
Source: Soc Psychiatry Psychiatr Epidemiol. 2023 Jan 19;58(5):779–94. doi: 10.1007/s00127-022-02377-w (PMC9848711; doi:10.1007/s00127-022-02377-w)
Supplement: Supplementary file 1 — Supplementary file1 (DOCX 133 KB) [file 127_2022_2377_MOESM1_ESM.docx]

**Supplement**

**Table S1: Sample distribution of cohort members across all covariates by cohort**

|  | **NCDS**  **%/mean**  (n=15,291) | **BCS70**  **%/mean**  (n=16,128) |
| --- | --- | --- |
| **Mental health trajectory** |  |  |
| No symptoms | 40.9 [40.2, 42.1] | 52.8 [51.5, 54.1] |
| Stable-low/Early adult-onset decreasing | 16.4 [15.7, 17.0] | 9.1 [8.4, 9.8] |
| Adult-onset decreasing | 10.8 [10.1, 11.6] | 11.0 [9.9, 12.2] |
| Midlife-onset decreasing / Midlife-onset increasing | 11.9 [11.1, 12.6] | 7.8 [7.2, 8.4] |
| Stable-high symptoms | 19.9 [19.1, 20.6] | 19.3 [18.1, 20.5] |
| **Early life factors** |  |  |
| **Sex** |  |  |
| Male | 50.6 [49.8, 51.4] | 51.1 [50.3, 51.8] |
| Female | 49.4 [48.6, 50.2] | 48.9 [48.2, 49.7] |
| **Breast Fed** |  |  |
| Yes | 67.9 [67.1, 68.7] | 36.7 [35.9, 37.6] |
| No | 32.1 [31.3, 32.9] | 63.3 [62.4, 64.1] |
| **Smoked during pregnancy** |  |  |
| Yes | 29.4 [28.6, 30.3] | 41.0 [40.2, 41.7] |
| No | 70.6 [69.7, 71.4] | 59.0 [58.3, 59.8] |
| **Gestation period (days)** | 280.8 [280.5, 281.0] | 281.3 [281.0, 281.6] |
| **Birthweight (grams)** | 3329.9 [3320.2, 3337.6] | 3302 [3293.7, 3310.3] |
| **Family characteristics** |  |  |
| **Parental social class (RGSC) (0)** |  |  |
| Professional | 4.6 [4.4, 4.9] | 4.3 [4.0, 4.7] |
| Managerial | 12.9 [12.3, 13.4] | 22.0 [21.2, 22.8] |
| Skilled non-manual | 10.1 [9.4, 10.6] | 10.0 [9.4, 10.6] |
| Skilled manual | 50.0 [49.0, 51.0] | 38.5 [37.6, 39.4] |
| Semi-skilled manual | 12.7 [12.1, 13.3] | 13.7 [13.0, 14.3] |
| Unskilled manual | 9.8 [9.2, 10.4] | 6.8 [6.3, 7.2] |
| Retired/unemployed | - | 4.7 [4.3, 5.1] |
| **Fathers education (post compulsory age)** |  |  |
| Yes | 23.0 [22.3, 23.8] | 19.9 [19.2, 20.5] |
| No | 77.0 [76.2, 77.7] | 80.1 [79.4, 80.8] |
| **Mothers education (post compulsory age)** |  |  |
| Yes | 25.0 [24.2, 25.7] | 17.7 [17.1, 18.3] |
| No | 75.0 [74.3, 75.8] | 82.3 [81.7, 82.9] |
| **Housing Tenure (5)** |  |  |
| Home owner | 42.0 [41.1, 42.8] | 55.3 [54.4, 56.2] |
| Other | 58.0 [57.2, 58.9] | 44.7 [43.8, 45.6] |
| **Access to house amenities** |  |  |
| Sole | 80.8 [80.0, 81.5] | 94.0 [93.4, 94.4] |
| Not sole | 19.2 [18.5, 20.0] | 6.0 [5.5, 6.6] |
| **Crowding (age 7/5)** |  |  |
| < 1 person per room | 57.1 [56.1, 57.9] | 35.9 [35.0, 36.8] |
| 1 to 1.49 person per room | 27.7 [26.9, 28.5] | 51.2 [50.4, 52.1] |
| 1.5 or more person per room | 15.3 [14.6, 15.9] | 12.9 [12.2, 13.5] |
| **Crowding (age 11/10)** |  |  |
| < 1 person per room | 60.5 [59.7, 61.3] | 30.3 [29.4, 21.2] |
| 1 to 1.49 person per room | 27.2 [26.4, 28.0] | 52.3 [51.3, 53.4] |
| 1.5 or more person per room | 12.3 [11.7, 12.9] | 17.4 [16.7, 18.1] |
| **Marital status** |  |  |
| Married | 96.1 [95.7, 96.4] | 92.4 [92.0. 92.9] |
| Other | 3.9 [3.6, 4.3] | 7.6 [7.1, 8.0] |
| **Read to child (age 7/5)** | 55.4 [54.4, 56.3] |  |
| **Maternal age at birth** | 27.5 [27.4, 27.6] | 25.9 [25.8, 26.0] |
| **Mother worked in first 5 years** |  |  |
| Yes | 29.4 [28.5, 30.2] | 30.9 [30.0, 31.7] |
| No | 70.6 [69.8, 71.4] | 69.1 [68.3, 70.0] |
| **Separated from child >1 month** |  |  |
| Yes | 12.0 [11.4, 12.6] | 5.6 [5.0, 6.1] |
| No | 88.0 [87.4, 88.6] | 94.4 [93.9, 95.0] |
| **Child health** |  |  |
| **Wet the bed at age 5** |  |  |
| Yes | 10.8 [10.3, 11.3] | 22.5 [21.8, 23.2] |
| No | 89.2 [88.7, 89.7] | 77.5 [76.7, 78.2] |
| **Had any medical conditions** |  |  |
| None | 56.6 [55.7, 57.4] | 46.4 [45.4, 47.4] |
| One | 27.7 [27.0, 28.6] | 29.9 [29.0, 30.8] |
| Two or more | 15.6 [15.0, 16.2] | 23.7 [22.8, 24.5] |

**Table S2a: Relative risk (RR) of change in financial and employment circumstances associated with pre-pandemic psychological distress trajectories in the NCDS and BCS70 during the COVID-19 pandemic**

**(Impute and delete’ method [1]).**

|  | NCDS | | | | BCS70 | | | |
| --- | --- | --- | --- | --- | --- | --- | --- | --- |
| Outcomes during COVID  Ref: No symptoms | Stable-low | Adult-onset | Midlife-onset | Stable-high | Early adult-onset | Adult onset | Midlife-onset | Stable-high |
| During COVID-19 pandemic  Current financial situation (May 2020)  Better off  A little worse off  Much worse off | 0.98  [0.78, 1.22]  1.07  [0.88, 1.31]  1.41  [1.05, 1.90] | 1.02  [0.76, 1.37]  1.04  [0.79, 1.35]  1.64  [1.11, 2.42] | 0.92  [0.70, 1.21]  1.06  [0.84, 1.34]  1.14  [0.78, 1.66] | 0.82  [0.61, 1.21]  1.02  [0.80, 1.30]  1.35  [0.93, 1.96] | 0.86  [0.65, 1.15]  0.79  [0.59, 1.05]  1.15  [0.80, 1.66] | 1.27  [0.93, 1.74]  1.28  [0.94, 1.73]  1.34  [0.89, 2.03] | 1.07  [0.79, 1.44]  1.27  [0.95, 1.69]  1.31  [0.88, 1.94] | 1.06  [0.78, 1.46]  1.40  [1.06, 1.86]  1.94  [1.34, 2.80] |
| Current financial situation (Sep/Oct 2020)  Better off  A little worse off  Much worse off | 0.87  [0.72, 1.06]  1.25  [1.02, 1.54]  1.35  [1.02, 1.80] | 0.90  [0.69, 1.19]  1.18  [0.90, 1.54]  1.46  [1.02, 2.09] | 0.80  [0.62, 1.02]  1.27  [1.01, 1.60]  1.20  [0.85, 1.70] | 0.93  [0.73, 1.19]  1.45  [1.15, 1.83]  1.79  [1.30, 2.47] | 0.73  [0.57, 0.94]  1.13  [0.88, 1.44]  1.21  [0.83, 1.78] | 1.02  [0.78, 1.33]  1.05  [0.79, 1.41]  1.86  [1.27, 2.73] | 0.87  [0.67, 1.14]  1.08  [0.82, 1.42]  1.71  [1.18, 2.48] | 0.78  [0.60, 1.01]  1.19  [0.92, 1.53]  2.33  [1.68, 3.23] |
| Current financial situation (Feb/March 2021)  Better off  A little worse off  Much worse off | 0.94  [0.79, 1.11]  1.02  [0.84, 1.22]  1.15  [0.89, 1.48] | 1.10  [0.88, 1.39]  1.21  [0.96, 1.54]  1.56  [1.13, 2.15] | 0.88  [0.71, 1.09]  0.88  [0.70, 1.12]  1.40  [1.06, 1.86] | 0.76  [0.60, 0.95]  1.21  [0.98, 1.49]  1.73  [1.32, 2.27] | 0.93  [0.74, 1.17]  1.32  [1.02, 1.69]  1.34  [0.98, 1.83] | 0.89  [0.70, 1.14]  1.21  [0.91, 1.60]  1.45  [1.03, 2.03] | 0.94  [0.74, 1.19]  1.13  [0.86, 1.49]  1.74  [1.27, 2.38] | 0.81  [0.64, 1.04]  1.37  [1.07, 1.75]  2.06  [1.54, 2.76] |
| Change in employment status (March-May 2020)  Work-furlough  Work-not work | 1.11  [0.89, 1.38]  1.25  [0.77, 2.03] | 0.92  [0.67, 1.27]  0.82  [0.38, 1.75] | 0.85  [0.64, 1.13]  1.46  [0.83, 2.56] | 1.20  [0.90, 1.59]  1.62  [0.90, 2.91] | 1.18  [0.91, 1.54]  0.84  [0.34, 2.04] | 1.04  [0.78, 1.39]  1.57  [0.73, 3.40] | 1.29  [0.98, 1.69]  0.98  [0.39, 2.49] | 1.28  [0.97, 1.70]  1.13  [0.47, 2.71] |
| Change in employment status (March-Sept/Oct 2020)  Work-furlough  Work-not work | 1.09  [0.79, 1.51]  1.40  [1.01, 1.94] | 1.01  [0.64, 1.61]  1.39  [0.88, 2.18] | 1.09  [0.73, 1.61]  1.23  [0.82, 1.84] | 1.39  [0.94, 2.05]  1.41  [0.93, 2.13] | 0.91  [0.55, 1.51]  1.06  [0.59, 1.89] | 2.05  [1.33, 3.16]  1.26  [0.68, 2.34] | 1.18  [0.70, 1.98]  1.32  [0.73, 2.41] | 1.62  [1.02, 2.57]  1.39  [0.79, 2.45] |
| Change in employment status (March-Feb/Mar 2021)  Work-furlough  Work-not work | 0.81  [0.62, 1.06]  1.10  [0.85, 1.44] | 1.43  [1.04, 1.95]  1.46  [1.04, 2.07] | 1.00  [0.74, 1.36]  1.11  [0.80, 1.53] | 1.58  [1.20, 2.09]  1.05  [0.80, 1.53] | 0.96  [0.71, 1.30]  1.36  [0.83, 2.23] | 1.41  [1.03, 1.91]  1.60  [0.95, 2.67] | 1.23  [0.91, 1.68]  1.48  [0.88, 2.49] | 1.18  [0.87, 1.61]  1.47  [0.87, 2.49] |

Current financial situation: NCDS: W1 n=4,914, W2 n=6,229, W3 n=6,757; BCS70: W1 n=3,902; W2 n=5,124; W3 n=5,548

Change in employment status: NCDS: W1 n=4,880, W2 n=5,997, W3 n=6,709; BCS70: W1 n=3,869; W2 n=4,931; W3 n=5,480

Reported as Relative Risk ratio (RRR), 95% confidence intervals (95% CIs)

Parameters are adjusted for sex, breastfed, mother smoked during pregnancy, gestation period, birthweight, parental social class at 0, parental education at 0, parental income, housing tenure at 7/5, access to house amenities at 7/5, total household income at 0, crowding at age 0, 7/5 and 11/10, parents marital status at 0, maternal age at birth, mother worked in first five years, separated from child for more than a month <age 5, read to at 7/5, CM wet the bed at 7/5, had any medical conditions at 7/5, Body Mass Index (BMI) at 11/10, and cognitive ability at 7/5 and 11/10.

**Table S2b: Relative risk (RR) of using methods to mitigate the economic shock associated with pre-pandemic psychological distress trajectories in the NCDS and BCS70 during the COVID-19 pandemic**

**(Impute and delete’ method [1]).**

|  |  | NCDS | |  |  | BCS70 | |  |
| --- | --- | --- | --- | --- | --- | --- | --- | --- |
| Outcomes during COVID  Ref: No symptoms | Stable-low | Adult-onset | Midlife-onset | Stable-high | Early adult-onset | Adult-onset | Midlife-onset | Stable-high |
| Any new benefit claims March 2020 – March 2021 | 0.98  [0.85, 1.13] | 1.12  [0.93, 1.33] | 1.16  [0.99, 1.36] | 1.20  [1.04, 1.40] | 1.15  [0.97, 1.35] | 1.20  [1.00, 1.42] | 1.38  [1.18, 1.61] | 1.39  [1.20, 1.61] |
| Payment holidays since March 2020 – March 2021 | 1.07  [0.83, 1.39] | 1.38  [1.01, 1.89] | 1.24  [0.91, 1.69] | 1.58  [1.20, 2.08] | 1.35  [1.13, 1.61] | 1.08  [0.87, 1.35] | 1.19  [0.98, 1.44] | 1.27  [1.06, 1.53] |
| Increased financial help from family and friends | 1.81  [1.15, 2.84] | 2.15  [1.27, 3.65] | 1.70  [1.01, 2.87] | 2.61  [1.63, 4.12] | 1.21  [0.77, 1.90] | 1.75  [1.17, 2.61] | 1.64  [1.10, 2.44] | 1.76  [1.22, 2.54] |
| Other methods for mitigating economic shock  Spending less | 1.13  [0.98, 1.30] | 1.27  [1.06, 1.52] | 1.02  [0.86, 1.22] | 1.23  [1.05, 1.45] | 1.05  [0.88, 1.27] | 1.24  [1.03, 1.49] | 1.32  [1.12, 1.56] | 1.40  [1.20, 1.64] |
| Using savings | 1.01  [0.84, 1.20] | 1.13  [0.91, 1.42] | 1.25  [1.03, 1.51] | 1.31  [1.08, 1.58] | 1.22  [0.96, 1.55] | 1.59  [1.27, 1.99] | 1.50  [1.21, 1.87] | 1.65  [1.34, 2.02] |
| Borrowing bank or credit card | 1.11  [0.66, 1.90] | 1.58  [0.87, 2.89] | 1.13  [0.61, 2.09] | 2.14  [1.29, 3.55] | 1.72  [1.17, 2.53] | 1.72  [1.14, 2.59] | 1.13  [0.71, 1.80] | 1.96  [1.36, 2.83] |
| Borrowing friends and family | 1.19  [0.51, 2.77] | 1.94  [0.79, 4.73] | 1.25  [0.50, 3.12] | 2.51  [1.15, 5.49] | 1.32  [0.70, 2.46] | 1.22  [0.60, 2.47] | 2.06  [1.20, 3.54] | 3.43  [2.21, 5.32] |
| On universal credit (Jan 2020-March 2021) | 1.01  [0.72, 1.42] | 1.44  [1.00, 2.07] | 1.33  [0.93, 1.89] | 1.88  [1.39, 2.55] | 1.13  [0.82, 1.58] | 1.73  [1.30, 2.31] | 1.41  [1.03, 1.92] | 1.65  [1.27, 2.15] |

New benefits NCDS n=7,517, BCS70 n=6,704; Payment holidays NCDS n=7,721, BCS70 n=6,712; Financial help family/friends n=7,282, BCS70 n=6,332

Other methods mitigating economic shock NCDS n=7,111, BCS70 n=6,074; Universal credit: NCDS n=7,533, BCS70 n=6,716

Reported as Relative Risk (RR), 95% confidence intervals (95% CIs)

Parameters are adjusted for sex, breastfed, mother smoked during pregnancy, gestation period, birthweight, parental social class at 0, parental education at 0, parental income, housing tenure at 7/5, access to house amenities at 7/5, total household income at 0, crowding at age 0, 7/5 and 11/10, parents marital status at 0, maternal age at birth, mother worked in first five years, separated from child for more than a month <age 5, read to at 7/5, CM wet the bed at 7/5, had any medical conditions at 7/5, Body Mass Index (BMI) at 11/10, and cognitive ability at 7/5 and 11/10.

**Table S3a-S3c: Details on latent class analysis**

The analytic sample for the latent class analysis (LCA) included all individuals with at least four assessments of psychological distress from adolescence to midlife, and who were alive and had not emigrated (n=11,579 in the NCDS and n=10,236 in the BCS70) by the age of 50 and 46 respectively. Missing data within the LCA was modelled with Full Information Maximum Likelihood [2].

As would be expected in the general population, many cohort members had no or few symptoms of psychological distress. As a result, we applied a four-category ordinal variable on the basis of grouping factor scores distributions of the latent measures to each of the five time points in each cohort. The first group consisted of individuals from the 1^st^ to 50^th^ percentile on the factor scores (i.e. lack of or no symptoms), the second included individuals from the 51^st^ to the 75^th^ percentile (i.e. occasional symptoms), the third group included individuals from the 76^th^ and 90^th^ percentile (i.e. moderate symptoms), and the fourth group individuals from the 91^st^ to 100^th^ percentile (i.e. severe symptoms).

As latent classes are unobserved and the appropriate number of latent classes initially unknown, we explored models with three to seven classes, to identify the most parsimonious model (s-Table 3a and 3b). We used several indices of goodness of fit to compare models with different number of classes; a bootstrap p value for the likelihood ratio $x^{2}$ test (p<.01 indicating good fit), Bayesian Information Criteria (lowest value has best fit), Akaike’s Information Criteria (lowest value has best fit), and the Lo-Mendell-Rubin likelihood ratio bootstrap p value to compare whether an additional latent class improves model fit. In addition, we used Entropy a composite that indicates the overall ability of a mixture model to return well-separated profiles [3]. All Latent mixture modelling was conducted using MPLUS v8.2(Muthén and Muthén).

**Table S3a: Indices of model fit for a Latent Profile Model in NCDS**

|  | 3 classes | 4 classes | 5 classes | 6 classes | 7 classes |
| --- | --- | --- | --- | --- | --- |
| Likelihood ratio bootstrap p valueᵅ | <.001 | <.001 | <.01 | <.001 | <.001 |
| Bayesian Information Criteriaᵇ | 126,305 | 123,017 | 121,283 | 121,313 | 120.295 |
| Akaike’s Information Criteriaᶜ | 126,144 | 122,811 | 121,033 | 121.020 | 119,957 |
| Lo-Mendell-Rubin bootstrap p value of likelihood ratio test ᵈ | <.001 | <.001 | <.001 | .427 | <.001 |
| Entropyᵉ | .830 | .836 | .903 | .813 | .810 |

ᵅ >.01 indicates good fit. ᵇlowest value indicate best fit. ᶜ lowest value indicate best fit. ᵈ comparing current to previous model, p<.05 indicates addition of this class significantly improves fit. ᵉhigher entropy (up to perfect classification at 1) indicate a better fit

**Table S3b: Indices of model fit for a Latent Profile Model in BCS70**

|  | 3 classes | 4 classes | 5 classes | 6 classes | 7 classes |
| --- | --- | --- | --- | --- | --- |
| Likelihood ratio bootstrap p valueᵅ | <.001 | <.001 | <.001 | <.001 | <.001 |
| Bayesian Information Criteriaᵇ | 94,278 | 93,621 | 92,799 | 92,192 | 91,557 |
| Akaike’s Information Criteriaᶜ | 94,277 | 93,419 | 92,554 | 91,903 | 91,225 |
| Lo-Mendell-Rubin bootstrap p value of likelihood ratio test ᵈ | <.001 | <.001 | <.05 | <.001 | <.001 |
| Entropyᵉ | .788 | .772 | .782 | .777 | .744 |

ᵅ >.01 indicates good fit. ᵇlowest value indicate best fit. ᶜ lowest value indicate best fit. ᵈ comparing current to previous model, p<.05 indicates addition of this class significantly improves fit. ᵉhigher entropy (up to perfect classification at 1) indicate a better fit

In the NCDS, when comparing 5 to 6 classes the Lo-Mendell-Rubin bootstrap p value of likelihood ratio test indicated that the addition of another latent class did not significantly improve model fit. In addition, the entropy in the 5 class model increases to 0.903 from the 4 class model and declines again from the 5 to 6 class solution. In the BCS70, the Lo-Mendell-Rubin bootstrap p value of likelihood ratio test was significant for all classes and the entropy was similar across all models, albeit declining from class 5 0.782 to 0.744 in the 7 class model. However, in the 6 class model, the prevalence of the 6^th^ class was low (3.1%), along with the probability of classification to the group 0.540. In both cohorts we therefore selected the 5 class model as the most parsimonious summary of psychological distress from adolescence to midlife.

The five longitudinal groups were not identical in the two cohorts, most of the groupings were very similar. In both cohorts, the largest group had ‘no symptoms’ (NCDS n = 5057 (43.7%), BCS70 n = 5821, (56.9%), There was also a group with persistent and repeated severe symptoms ‘stable-high’ (NCDS n=1749 (15.1%), BCS70 n=1275 (12.5%), and a group with adult onset and favourable outcomes ‘adult-onset’ decreasing (NCDS=1172 (10.1%), BCS70=1114 (10.9%)). Both cohorts also had a group with symptoms developing in midlife; however for this group in the NCDS by their 50’s psychological distress was decreasing ‘midlife-onset decreasing’ n=1375, 11.9%) and in the BCS70 the onset of symptoms were severe and increasing ‘midlife-onset decreasing’ n= 961, 9.4%). The final group in the NCDS was repeated minor symptoms ‘stable-low symptoms’ (n=2226, 19.2%), while in the BCS70 the fifth grouping had early adult onset and more favorable outcomes ‘early adult-onset’ decreasing (BCS70=1065, 10.4%). These differences may be due to cohort effects, for example the period 1990-1993 was marked by a long recession when the BCS70 cohort members would have been new entrants to the labor market,[4] perhaps resulting in increased levels of distress for some.

For both studies we used different measures and reporters (the parent at age 16 and self-report in adulthood) of psychological distress at age 16, compared to adulthood. We acknowledge that correlations between parent and self-report tend to be low [5]. As self-report Malaise Inventory was available at age 16 in the BCS70 only, we reran the models and observed the same trajectories for the 5 classes identified using the original measures and a difference in trajectory classification of only 2.9% (Table S3c).

**Table 3c: Classification of longitudinal classes comparing CBQ with Malaise Inventory at age 16**

|  |  | Longitudinal classes with CBQ at age 16 | | | | | |
| --- | --- | --- | --- | --- | --- | --- | --- |
|  |  | NS | EAD | AOD | MOI | SHS | Total |
|  | NS | 5668 | 36 | 19 | 22 | 0 | 5745 |
| Mala |  | 97.8% | 3.2% | 1.8% | 2.3% | 0.0% | 56.3% |
|  | EA**D** | 66 | 1055 | 0 | 3 | 16 | 1140 |
|  |  | 1.1% | 94.7% | 0.0% | 0.3% | 1.3% | 11.2% |
|  | AOD | 20 | 0 | 1039 | 8 | 16 | 1083 |
|  |  | 0.3% | 0.0% | 97.6% | 0.8% | 1.3% | 10.6% |
|  | MOI | 42 | 0 | 2 | 919 | 14 | 977 |
|  |  | 0.7% | 0.0% | 0.2% | 95.6% | 1.1% | 9.6% |
|  | SHS | 0 | 23 | 5 | 9 | 1229 | 1266 |
|  |  | 0.0% | 2.1% | 0.5% | 0.9% | 96.4% | 12.4% |
|  |  | 5796 | 1114 | 1065 | 961 | 1275 | 10211 |
|  |  | 100.0% | 100.0% | 100.0% | 100.0% | 100.0% | 100.0% |
|  | Total | 56.8% | 10.9% | 10.4% | 9.4% | 12.4% | 100.0% |

NS (No symptoms), EAD (Early adult-onset decreasing), AOD (Adult-onset decreasing), MOI (Midlife-onset increasing), SHS (Stable-high symptoms).

**Table S4: Details of covariates**

| Variable | Age of cohort member (CM) | Description |
| --- | --- | --- |
| Sex | 0 | Male=0 (ref) Female =1 |
| Maternal age at birth | 0 | Maternal age at birth in years |
| Breast-fed or not | 0 | Whether the mother ever breastfed the infant (1) or not (0) |
| Smoked during pregnancy | 0 | Whether during pregnancy the mother smoked more than 1 cigarette daily (1), or not (0) |
| Gestation period | 0 | Gestation period in days |
| Birthweight | 0 | Birthweight recorded in grams |
| Parental social Class | 0 | Occupation of the father was coded according to the Registrar General’s classification. Participant’s current or most recent jobs were classified as: 1 professional, 2 managerial and technical, 3 skilled non-manual 4 skilled manual, 5 partly-skilled manual, and 6 unskilled. |
| Father’s education | 0 | Father stayed on at school after the minimum school leaving age, yes (1) no (0). |
| Mother’s education | 0 | Mother stayed on at school after the minimum school leaving age, yes (1) no (0). |
| House tenure | 7 in NCDS and 5 in BCS70 | Whether the CM’s parents were home owners (1), or not (0). |
| Access to house amenities | 7 in NCDS and 5 in BCS70 | Whether the family had sole access to the following household amenities; bathroom, indoor WC, hot water, and kitchen (1) or not (0) |
| Total household income | 0 | Quintiles |
| Crowding | 0, 7, 11 in NCDS and 0, 5, 10 in BCS70 | Number of persons per room (excluding kitchen, toilet, and bathroom), up to 1 (0), 1 to 1.49 (1) and 1.5 or over (2). |
| Marital status | 0 | Whether parents were married (1) or not (0). |
| Read to child every week | 7 in NCDS and 5 in BCS70 | CM read to at least once a week, yes (1) no (0). |
| Mother worked before CM went to school | 7 in NCDS and 5 in BCS70 | Whether the mother worked full-time or part time before the child went to school, yes (1) no (0). (In the NCDS, mothers were asked whether they had paid work outside the home since CM’s birth and before child started school. In the BCS70, (at age 5) the mother was asked if they had a regular full-time or part-time job out of the home since the time of CM’s birth). |
| Separated with CM for more than one month | 7 in NCDS and 5 in BCS70 | Whether the mother has been separated from CM for one month or more, yes (1) no (0). |
| Bedwetting | 7 in NCDS58 and 5 in BCS70 | In the NCDS, whether CM has wet the bed since age 5, yes (1) no (0). In the BCS70, at age 5, does the CM wet the bed, yes (1) no (0). |
| Medical conditions | 7 in NCDS and 5 in BCS70 | Whether the CM had any of the following medical conditions; eczema, hay fever and sneezing, ear discharge, sore throats, bronchitis, pneumonia, hearing difficulty. The medical conditions were recoded into, none (0), one (1), two or more (2). |
| BMI | 11 in NCDS and 10 in BCS70 | Height and weight were measured by trained medical personnel using standard protocols at age 11 in the NCDS and at age 10 in BCS70. BMI was standardised, with a mean of 0 and a standard deviation of 1. |
| Cognitive ability | 7, 11 in NCDS and 5, 10 in BCS70 | In the NCDS at age 7, CM’s completed the Southgate Group Reading Test, Problematic Arithmetic Test, Copy Design Test and Human Figure Drawing; at age 11 they completed a General Ability Test (both verbal and non-verbal), a Reading Comprehension Test, a Mathematics Test and Copy designs Test. In the BCS70 at age 5 CM’s completed the English Picture Vocabulary Test, Copy Designs Test, Human Figure Drawing and Complete a Profile Test; at age 10 CM’s completed four tests from the British Ability Scales, Word Similarities, Word definitions, Recall of Digits and Matrices [6]. All tests were standardised, with a mean of 0 and standard deviation of 1. Within each age group and cohort, a Principal Component Analysis (PCA) was conducted to extract the common variance across all the cognitive tests and a single component predicted representing a general ability score. |

**Table S5:** **Predicted probability of changes in financial circumstance and employment outcomes associated with psychological distress trajectories in the NCDS and BCS70 at different stages during the pandemic**

|  |  |  | NCDS |  |  |  |  | BCS70 |  |  |
| --- | --- | --- | --- | --- | --- | --- | --- | --- | --- | --- |
| Outcomes pre and during COVID | No symptoms  % | Stable-low  % | Adult-onset  % | Midlife-onset  % | Stable-high  % | No symptoms  % | Early adult-onset  % | Adult-onset  % | Midlife-onset  % | Stable-high  % |
| During COVID-19 pandemic  Current financial situation (May 2020)  Better off  About the same  A little worse off  Much worse off | 11.6  [10.1, 13.2]  52.9  [49.4, 56.3]  24.8  [21.8, 27.7]  10.7  [8.0, 13.4] | 11.3  [8.9, 13.6]  51.1  [46.6, 55.4]  25.2  [21.8, 28.6]  12.4  [8.8, 16.1] | 10.8  [8.2, 13.4]  49.1  [43.4, 54.8]  24.0  [18.3, 29.7]  16.1  [11.9, 20.3] | 10.5  [8.0, 13.0]  51.2  [45.9, 56.6]  26.3  [21.1, 31.4]  12.0  [8.3, 15.7] | 8.0  [5.6, 10.4]  49.8  [42.8, 56.7]  25.2  [20.8, 29.6]  17.0  [11.3, 22.7] | 18.3  [15.9, 20.6]  44.3  [41.0, 47.7]  24.5  [21.8, 27.3]  12.9  [10.7, 15.0] | 16.8  [13.6, 20.1]  46.7  [40.8, 52.7]  20.6  [6.0, 25.2]  15.8  [11.8, 19.9] | 19.4  [15.5, 23.2]  38.2  [32.3, 44.0]  28.2  [21.8, 34.6]  14.3  [9.4, 19.1] | 17.9  [14.6, 21.2]  40.5  [34.9, 46.2]  27.7  [22.7, 32.7]  13.9  [9.3, 18.5] | 15.3  [11.6, 19.0]  36.8  [31.0, 42.5]  28.2  [23.0, 33.4]  19.7  [15.2, 24.3] |
| Current financial situation (Sep/Oct 2020)  Better off  About the same  A little worse off  Much worse off | 13.1  [11.5, 14.6]  61.9  [59.0, 64.9]  15.8  [13.3, 18.2]  9.2  [7.0, 11.4] | 11.2  [9.6, 12.9]  60.2  [56.4, 64.0]  18.3  [15.6, 21.0]  10.3  [7.9, 12.7] | 11.1  [8.6, 13.5]  58.4  [53.8, 62.9]  17.9  [14.6, 21.2]  12.7  [9.5, 15.8] | 10.2  [8.2, 12.2]  60.5  [56.6, 64.4]  18.9  [15.4, 22.3]  10.4  [7.0, 13.9] | 9.6  [7.6, 11.5]  54.1  [49.1, 59.2]  21.0  [17.6, 24.4]  15.3  [10.4, 20.1] | 18.6  [16.7, 20.4]  55.0  [52.2, 57.9]  18.5  [15.5, 21.4]  7.9  [6.0, 9.8] | 14.4  [11.7, 17.1]  54.9  [50.6, 59.3]  20.8  [17.5, 24.1]  9.8  [6.6, 13.0] | 17.2  [13.6, 20.8]  51.0  [45.1, 56.8]  17.4  [13.6, 21.2]  14.4  [10.0, 18.9] | 16.1  [13.1, 19.2]  52.4  [47.2, 57.6]  18.9  [14.5, 23.2]  12.6  [9.6, 15.6] | 12.7  [9.7, 15.6]  50.8  [46.6, 55.1]  18.8  [14.7, 22.8]  17.8  [12.7, 22.8] |
| Current financial situation (Feb/March 2021)  Better off  About the same  A little worse off  Much worse off | 18.0  [16.5, 19.6]  53.0  [50.4, 55.6]  18.4  [16.0, 20.7]  10.6  [8.7, 12.4] | 17.4  [16.5, 19.6]  53.8  [50.8, 56.8]  18.2  [15.7, 20.8]  10.6  [8.7, 12.4] | 18.2  [15.2, 21.3]  47.5  [43.4, 51.6]  19.4  [15.3, 23.4]  14.9  [11.4, 18.3] | 15.9  [13.7, 18.2]  53.3  [49.4, 57.3]  15.5  [12.6, 18.4]  15.2  [12.6, 17.8] | 12.6  [9.83, 15.4]  47.4  [44.3, 50.6]  20.0  [16.6, 23.5]  19.9  [16.0, 23.8] | 23.1  [21.2, 25.0]  50.4  [47.3, 53.6]  16.7  [14.6, 18.8]  9.7  [7.8, 11.6] | 20.8  [17.2, 24.4]  47.2  [42.9, 51.4]  20.0  [15.4, 24.4]  12.0  [9.2, 14.9] | 19.4  [16.1, 22.6]  48.3  [43.7, 53.0]  19.7  [15.5, 24.0]  12.6  [9.4, 15.7] | 20.6  [16.8, 24.4]  46.6  [41.4, 51.7]  17.1  [13.7, 20.5]  15.7  [11.8, 19.6] | 16.5  [13.4, 19.7]  45.7  [40.6, 50.9]  19.7  [16.5, 23.0]  18.0  [14.3, 21.7] |
| Change in employment status:  (March-May 2020)  Work-work  Work-furlough  Work-not work  Other | 33.9  [30.1, 37.8]  25.3  [21.3, 29.3]  3.8  [2.0, 5.6]  36.9  [33.3, 40.6] | 32.8  [29.7, 35.9]  25.2  [21.3, 29.1]  4.2  [2.2, 6.3]  37.8  [33.4, 42.1] | 33.2  [27.8, 38.7]  23.3  [17.3, 29.4]  3.0  [0.6, 5.4]  40.4  [35.3, 45.6] | 32.2  [27.6, 36.7]  21.6  [17.1, 26.2]  5.0  [2.2, 7.8]  41.2  [35.9, 46.4] | 25.0  [20.4, 29.6]  26.1  [21.0, 31.3]  5.7  [2.4, 9.0]  43.1  [37.6, 48.6] | 56.4  [53.2, 59.6]  26.4  [23.4, 29.6]  4.5  [1.3, 7.8]  12.5  [10.1, 15.0] | 52.7  [47.2, 58.1]  30.4  [24.2, 36.5]  3.1  [0.8, 5.4]  13.8  [10.0, 17.7] | 53.4  [48.3, 58.5]  26.1  [21.2, 31.0]  6.0  [1.6, 10.5]  14.5  [10.8, 18.1] | 47.2  [41.8, 52.7]  27.2  [22.0, 32.4]  3.4  [0.2, 6.6]  22.1  [16.9, 27.4] | 42.6  [37.7, 47.5]  24.8  [20.3, 29.3]  4.5  [1.4, 7.5]  28.1  [23.1, 33.1] |
| Change in employment status (March-Sept/Oct 2020)  Work-work  Work-furlough  Work-not work  Other | 50.4  [47.8, 52.9]  6.7  [4.9, 8.5]  4.4  [3.4, 5.5]  38.5  [35.9, 41.1] | 48.0  [44.7, 51.2]  6.7  [4.4, 9.0]  5.8  [4.3, 7.3]  39.5  [36.0, 43.0] | 47.7  [43.0, 52.3]  6.9  [4.1, 9.8]  5.7  [3.4, 7.8]  39.8  [35.3, 44.2] | 45.9  [42.9, 48.9]  6.9  [4.8, 9.1]  5.0  [3.3, 6.6]  42.2  [38.6, 45.8] | 39.6  [35.7, 43.4]  8.6  [5.1, 12.1]  5.2  [2.9, 7.6]  46.6  [41.6, 51.5] | 76.6  [74.4, 78.9]  4.7  [3.5, 5.9]  4.7  [3.3, 6.0]  14.0  [12.1, 15.9] | 75.5  [71.5, 79.5]  3.9  [2.4, 5.4]  4.7  [1.8, 7.6]  15.8  [12.9, 18.8] | 67.3  [62.3, 72.4]  8.3  [5.1, 11.5]  5.1  [2.5, 7.7]  19.3  [15.4, 23.1] | 68.0  [63.8, 72.2]  5.1  [2.7, 7.5]  4.6  [2.6, 6.6]  22.2  [18.8, 25.7] | 58.9  [54.0, 63.9]  6.6  [3.5, 9.6]  6.2  [2.7, 9.6]  28.4  [24.3, 32.4] |
| Change in employment status (March-Feb/Mar 2021)  Work-work  Work-furlough  Work-not work  Other | 43.6  [41.2, 45.9]  11.7  [10.2, 13.1]  7.6  [6.2, 9.0]  37.1  [35.0, 39.2] | 44.5  [41.9, 47.1]  8.9  [7.0, 10.8]  8.3  [6.8, 9.8]  38.2  [35.5, 41.0] | 37.2  [33.4, 41.0]  14.6  [10.3, 18.8]  9.4  [6.7, 12.0]  38.9  [34.7, 43.0] | 40.8  [37.4, 44.3]  11.3  [8.7, 13.9]  8.1  [6.1, 10.1]  39.7  [36.2, 43.3] | 30.1  [26.6, 33.6]  16.1  [12.3, 19.9]  6.8  [4.4, 9.2]  47.0  [42.9, 51.2] | 70.1  [67.3, 72.9]  13.0  [11.2, 14.8]  3.6  [2.6, 4.7]  13.2  [11.0, 15.4] | 70.0  [66.5, 73.4]  11.4  [8.9, 14.0]  4.8  [2.7, 7.0]  13.8  [10.7, 16.8] | 62.4  [57.7, 67.1]  15.3  [12.1, 18.5]  5.2  [2.3, 8.1]  17.1  [13.9, 20.3] | 60.6  [56.8, 64.4]  13.1  [10.6, 15.6]  4.5  [2.3, 6.8]  21.7  [18.3, 25.2] | 55.6  [50.8, 60.4]  11.2  [9.0, 14.4]  5.6  [3.4, 7.8]  27.1  [23.7, 30.4] |

**Table S6:** **Predicted probability of using methods to mitigate the economic shock associated with psychological distress trajectories in the NCDS and BCS70 at**

**during the COVID-19 pandemic**

|  |  |  | | NCDS | |  | |  | |  |  | | BCS70 | |  | |  | |
| --- | --- | --- | --- | --- | --- | --- | --- | --- | --- | --- | --- | --- | --- | --- | --- | --- | --- | --- |
| Outcomes pre and during COVID | No symptoms  % | | Stable-low  % | | Adult-onset  % | | Midlife-onset  % | | Stable-high  % | No symptoms  % | | Early adult-onset  % | | Adult-onset  % | | Midlife-onset  % | | Stable-high  % |
| New benefit claims made since March 2020 | 18.7  [17.0, 20.4] | | 17.4  [15.3, 19.7] | | 21.3  [18.5, 24.4] | | 22.1  [19.3, 25.4] | | 24.3  [21.8, 27.0] | 15.7  [14.0, 17.6] | | 19.0  [15.9, 22.6] | | 19.6  [16.3, 23.7] | | 22.1  [18.7, 26.2] | | 22.5  [18.8, 27.0] |
| Payment holidays since March 2020 | 6.9  [5.7, 8.3] | | 6.6  [5.4, 8.2] | | 9.9  [7.3, 13.4] | | 9.2  [7.3, 11.7] | | 12.4  [10.4, 14.8] | 14.7  [12.8, 16.8] | | 19.7  [16.2, 24.0] | | 16.6  [12.6, 21.7] | | 17.0  [13.7, 21.0] | | 19.1  [16.1, 22.6] |
| Increased financial help from family and friends | 2.0  [1.5, 2.9] | | 3.0  [2.1, 4.3] | | 4.2  [2.8, 6.4] | | 3.7  [2.4, 5.6] | | 5.8  [3.9, 8.6] | 1.7  [1.2, 2.3] | | 2.3  [1.3, 3.9] | | 3.0  [1.9, 4.8] | | 2.9  [1.9, 4.4] | | 2.9  [1.7, 5.0] |
| Other methods for mitigating economic shock:  Spending less | 17.1  [15.2, 19.3] | | 18.3  [16.4, 20.5] | | 21.8  [18.6, 25.6] | | 18.1  [15.3, 21.5] | | 22.4  [18.9, 26.4] | 17.4  [15.4, 19.7] | | 18.6  [15.5, 22.3] | | 21.7  [17.4, 27.0] | | 22.9  [19.3, 27.2] | | 24.4  [21.2, 28.3] |
| Using savings | 13.7  [12.1, 15.6] | | 12.8  [11.2, 14.7] | | 16.0  [12.6, 20.2] | | 16.9  [14.3, 19.9] | | 20.0  [17.3, 23.0] | 11.3  [9.7, 13.0] | | 13.7  [10.7, 17.6] | | 17.9  [14.1, 22.8] | | 16.3  [13.3, 20.1] | | 18.0  [15.0, 21.7] |
| Borrowing bank or credit card | 1.8  [1.3, 2.6] | | 1.8  [1.1, 3.0] | | 3.0  [1.7, 5.2] | | 2.6  [1.6, 4.3] | | 4.7  [3.2, 7.1] | 3.9  [3.1, 5.0] | | 6.1  [4.3, 8.7] | | 6.4  [4.3, 9.8] | | 3.8  [2.4, 5.7] | | 7.3  [4.9, 11.0] |
| Borrowing friends and family | 1.1  [0.6, 2.0] | | 1.1  [0.4, 2.5] | | 2.2  [1.1, 4.2] | | 1.8  [0.8, 3.7] | | 2.7  [1.4, 5.1] | 2.7  [1.8, 3.8] | | 3.6  [1.9, 7.0] | | 2.6  [1.4, 5.0] | | 4.9  [3.1, 7.8] | | 7.8  [5.1, 11.9] |
| On Universal Credit (January 2020-March 2021) | 6.3  [5.1, 7.8] | | 5.3  [3.9, 7.2] | | 9.3  [7.4, 11.7] | | 9.2  [7.0, 12.0] | | 13.2  [10.6, 16.5] | 5.9  [4.9, 7.1] | | 7.1  [5.3, 9.6] | | 10.3  [8.2, 12.9] | | 8.2  [6.0, 11.2] | | 9.8  [7.4, 12.9] |

**Table S7: Distribution of how cohort members were managing financially 3 months prior to COVID-19 by pre-pandemic psychological distress trajectories**

| Outcomes pre COVID | No symptoms  % | Stable-low  % | Adult-onset  % | Midlife-onset  % | Stable-high  % | Total  % | No symptoms  % | Early adult-onset  % | Adult-onset  % | Midlife-onset  % | Stable-high  % | Total  % |
| --- | --- | --- | --- | --- | --- | --- | --- | --- | --- | --- | --- | --- |
| 3 months prior to COVID-19:  Managing financially:  Living comfortably  Doing all right  Just about getting by  Finding it difficult | 52.5  [50.3, 54.6]  34.4  [32.1, 36.6]  10.0  [8.3, 11.7]  3.2  [1.7, 4.6] | 50.7  [48.2, 53.1]  36.7  [34.3, 39.0]  9.5  [7.5, 11.4]  3.2  [1.8, 4.5] | 40.3  [36.2, 44.5]  38.4  [32.9, 43.8]  16.0  [12.1, 19.9]  5.2  [2.5, 8.1] | 42.5  [39.3, 45.7]  36.1  [32.2, 40.0]  17.2  [13.4, 21.0]  4.2  [2.1, 6.3] | 26.1  [23.2, 28.9]  35.8  [31.3, 40.4]  29.2  [24.6, 33.8]  8.9  [5.2, 12.5] | 44.4  [42.8, 46.1)  35.7  [33.6, 37.8]  15.2  [13.3, 17.2]  4.7  [3.1, 6.2] | 42.6  [40.8, 44.4]  41.0  [38.7, 43.2]  12.9  [11.3, 14.4]  3.5  [2.4, 4.7] | 29.9  [26.4, 33.3]  43.1  [39.2, 47.0]  19.0  [15.0, 23.0]  8.0  [5.3, 10.8] | 30.2  [26.2, 34.2]  42.4  [38.2, 46.7]  19.5  [15.5, 23.6]  7.8  [5.4, 10.2] | 26.5  [22.8, 30.3]  41.0  [36.0, 45.9]  25.5  [20.9, 30.2]  7.0  [4.4, 9.5] | 15.7  [13.4, 18.1]  33.0  [28.4, 37.5]  34.3  [28.4, 40.1]  17.0  [11.0, 23.1] | 33.6  [32.3, 35.0]  39.8  [37.7, 41.9]  19.3  [17.0, 21.5]  7.3  [5.5, 9.1] |
| On benefits: | 17.7  [15.4, 20.0] | 15.2  [13.4, 17.1] | 26.0  [22.4, 29.6] | 26.1  [22.6, 29.7] | 40.7  [36.5, 44.9] | 23.8  [21.8, 25.7] | 18.9  [17.0, 20.8] | 25.0  [21.1, 28.8] | 27.1  [22.7, 31.5] | 31.3  [27.1, 35.6] | 46.9  [41.9, 51.9] | 26.7  [24.4, 29.0] |

**Table S8: Relative risk (RR) of managing financially and on benefits associated with pre-pandemic psychological distress trajectories in the NCDS and BCS70 3 months prior to the COVID-19 pandemic**

| Outcomes pre COVID  Ref: No symptoms | Stable-low  (RRR/RR) | Adult-onset | Midlife-onset | Stable-high | Early adult-onset | Adult-onset | Midlife-onset | Stable-high |
| --- | --- | --- | --- | --- | --- | --- | --- | --- |
| 3 months prior to COVID-19:  Managing financially: Ref – doing all right  Living comfortably  Just about getting by  Finding it difficult | 0.87  [0.76, 1.00]  0.92  [0.70, 1.21]  1.10  [0.64, 1.90] | 0.73  [0.62, 0.87]  1.33  [0.87, 2.02]  1.69  [0.99, 2.88] | 0.76  [0.64, 0.90]  1.61  [1.17, 2.21]  1.56  [0.86, 2.84] | 0.54  [0.46, 0.63]  2.61  [1.90, 3.58]  3.18  [2.04, 4.95] | 0.67  [0.56, 0.81]  1.35  [1.04, 1.75]  2.08  [1.35, 3.19] | 0.76  [0.59, 0.98]  1.33  [1.02, 1.74]  2.00  [1.32, 3.04] | 0.64  [0.50, 0.81]  1.94  [1.46, 2.58]  1.92  [1.26, 2.92] | 0.59  [0.49, 0.73]  2.68  [2.08, 3.43]  4.89  [3.12, 7.67] |
| On benefits: | 0.88  [0.77, 1.02] | 1.33  [1.11, 1.59] | 1.41  [1.23, 1.62] | 1.83  [1.61, 2.08] | 1.25  [1.09, 1.43] | 1.33  [1.16, 1.53] | 1.57  [1.38, 1.79] | 2.02  [1.80, 2.26] |

**Table S9: Relative risk (RR) of being worse off financially during the COVID-19 pandemic associated with pre-pandemic psychological distress trajectories in the NCDS and BCS70 stratified by financially struggling or comfortable 3 months prior to COVID-19**

| Outcomes during COVID-19  Ref: No symptoms | Stable-low | Adult-onset | Midlife-onset | Stable-high | Early adult-onset | Adult-onset | Midlife-onset | Stable-high |
| --- | --- | --- | --- | --- | --- | --- | --- | --- |
| 3 months prior to COVID-19:  Managing financially during COVID:  Wave 1:  Struggling  Comfortable | 1.12  [0.70, 1.80]  1.25  [0.90, 1.74] | 1.55  [0.90, 2.68]  1.54  [1.01, 2.34] | 0.98  [0.74, 2.41]  1.07  [0.68, 1.67] | 1.33  [1.90, 3.58]  1.33  [0.79, 2.23] | 1.06  [0.66, 1.71]  1.10  [0.77, 1.57] | 1.17  [0.63, 2.15]  1.23  [0.78, 1.94] | 1.01  [0.56, 1.80]  1.06  [0.71, 1.57] | 1.46  [0.88, 2.42]  1.64  [1.14, 2.37] |
| Wave 2:  Struggling | 1.10  [0.65, 1.90] | 1.24  [0.73, 2.08] | 0.87  [0.51, 1.47] | 1.18  [0.73, 1.91] | 1.10  [0.66, 1.83] | 1.66  [0.92, 3.01] | 1.25  [0.76, 2.04] | 1.67  [1.02, 2.74] |
| Comfortable | 1.18  [0.84, 1.66] | 1.36  [0.86, 2.14] | 1.06  [0.70, 1.60] | 1.47  [0.94, 2.30] | 1.06  [0.68, 1.67] | 1.87  [1.24, 2.81] | 1.48  [0.95, 2.29] | 1.82  [1.24, 2.67] |
| Wave 3:  Struggling | 1.07  [0.64, 1.77] | 1.43  [0.83, 2.46] | 1.25  [0.80, 1.94] | 1.61  [1.04, 2.50] | 1.17  [0.77, 1.79] | 1.27  [0.76, 2.13] | 1.46  [0.93, 2.27] | 1.53  [1.13, 2.07] |
| Comfortable | [0.77, 1.31] | 1.47  [1.06, 2.04] | 1.38  [1.00, 1.90] | 1.57  [1.00, 1.90] | 1.20  [0.81, 1.78] | 1.21  [0.88, 1.65] | 1.57  [1.01, 2.25] | 1.52  [1.08, 2.41] |
| Work-furlough (ref: work-work) |  |  |  |  |  |  |  |  |
| Wave 1:  Struggling  Comfortable | 1.03  [0.60, 1.76]  1.03  [0.80, 1.32] | 0.97  [0.53, 1.80]  0.89  [0.63, 1.27] | 0.83  [0.53, 1.30]  0.87  [0.65, 1.16] | 1.32  [0.85, 2.04]  1.26  [0.91, 1.75] | 1.16  [0.70, 1.92]  1.21  [0.91, 1.62] | 0.98  [0.62, 1.54]  1.03  [0.81, 1.32] | 1.35  [0.84, 2.16]  1.16  [0.88, 1.52] | 1.12  [0.75, 1.66]  1.23  [0.91, 1.65] |
| Wave 2:  Struggling  Comfortable | [0.54, 1.89]  1.07  [0.74, 1.56] | 1.05  [0.52, 2.14]  1.02  [0.61, 1.69] | 0.91  [0.49, 1.70]  1.13  [0.77, 1.67] | 1.38  [0.80, 2.37]  1.34  [0.84, 2.13] | 0.71  [0.30, 1.65]  0.84  [0.50, 1.39] | 1.76  [0.84, 3.70]  2.01  [1.26, 3.21] | 0.89  [0.38, 2.05]  1.25  [0.77, 2.04] | 1.52  [0.80, 2.87]  1.60  [1.00,2.55] |
| Wave 3:  Struggling  Comfortable | 0.60  [0.35, 1.03]  0.76  [0.56, 1.03] | 1.47  [0.87, 2.49]  1.30  [0.95, 1.79] | 1.10  [0.63, 1.92]  0.89 (  [0.66, 1.19] | 1.81  [1.11, 2.96]  1.50  [1.09, 2.08] | 0.69  [0.37, 1.29]  0.89  [0.65, 1.22] | 1.25  [0.78, 2.01]  1.33  [0.99, 1.79] | 1.01  [0.56, 1.82]  1.19  [0.86, 1.66] | 1.08  [0.69, 1.68]  1.04  [0.73, 1.49] |
| Work-unemployed (ref: work-work) |  |  |  |  |  |  |  |  |
| Wave 1:  Struggling  Comfortable | 1.22  [0.52, 2.85]  1.15  [0.67, 2.00] | 0.74  [0.23, 2.38]  0.70  [0.32, 1.51] | 1.13  [0.47, 2.69]  1.33  [0.75, 2.34] | 1.54  [0.71, 3.33]  1.71  [0.91, 3.23] | 0.65  [0.23, 1.81]  0.65  [0.28, 1.54] | 1.20  [0.51, 2.79]  1.42  [0.59, 3.43] | 0.69  [0.18, 2.66]  0.83  [0.30, 2.29] | 0.99  [0.42, 2.37]  1.16  [0.54, 2.47] |
| Wave 2:  Struggling  Comfortable | 1.75  [0.68, 4.53]  1.34  [0.94, 1.93] | 1.32  [0.51, 3.39]  1.36  [0.82, 2.17] | 0.96  [0.41, 2.26]  1.28  [0.90, 1.82] | 1.35  [0.48, 3.80]  1.51  [0.95, 2.40] | 0.80  [0.34, 1.87]  0.98  [0.54, 1.78] | 1.15  [0.55, 2.40]  1.17  [0.67, 1.86] | 0.84  [0.41, 1.73]  1.11  [0.67, 1.86] | 1.38  [0.67, 2.85]  1.47  [0.86, 2.50] |
| Wave 3:  Struggling  Comfortable | 1.19  [0.60, 2.34]  1.06  [0.82, 1.36] | 1.62  [0.77, 3.43]  1.53  [1.15, 2.03] | 1.12  [0.56, 2.25]  1.14  [0.81, 1.60] | 1.20  [0.63, 2.28]  1.25  [0.84, 1.84] | 1.23  [0.57, 2.65]  1.21  [0.64, 2.29] | 1.38  [0.55, 3.47]  1.59  [0.89, 2.82] | 1.22  [0.54, 2.77]  1.34  [0.72, 2.50] | 1.63  [0.79, 3.35]  1.67  [0.87, 3.17] |

**Supplementary references**

1. Von Hippel PT (2007) Regression with missing Ys: An improved strategy for analyzing multiply imputed data. *Sociological Methodology*  37: 83-117.
2. Enders CE (2010) Applied missing data analysis. New York: Guilford.
3. Celeux G, Soromenho G (1996) An entropy criterion for assessing the number of clusters in a mixture model. Journal of classification 13(2):195-212.
4. Sullivan A, Brown M, Bann D (2015) Guest Editorial: Generation X enters middle age. Longitudinal and Life Course Studies 6(2):120-30.
5. Collishaw S, Goodman R, Ford T, Rabe-Hesketh S, Pickles A (2009) How far are associations between child, family and community factors and child psychopathology informant‐specific and informant‐general?. Journal of Child Psychology and Psychiatry 50(5):571-80.
6. Moulton V, McElroy E, Richards M, Fitzsimons E, Northstone K, Conti G, Ploubidis GB, Sullivan A, O’Neill D (2020) A guide to the cognitive measures in five British birth cohort studies. London, UK: CLOSER.
